# Targeting the human gut microbiome: a comparative review of probiotics, prebiotics, synbiotics, and postbiotics

**DOI:** 10.1016/j.jare.2025.12.032

**Published:** 2025-12-27

**Authors:** Chunying Sun, Jingwen Zhu, Xueyuan Sun, Zhidong Zhang, Yantong Sun, Yan Jin, Tao Wu

**Affiliations:** State Key Laboratory of Food Nutrition and Safety, Food Biotechnology Engineering Research Center of Ministry of Education, College of Food Science and Engineering, Tianjin University of Science & Technology, Tianjin 300457, China

**Keywords:** Human microbiome, Probiotics, Prebiotics, Synbiotics, Postbiotics

## Abstract

**Background:**

The human gut microbiome plays a central role in regulating host health, serving as a core hub for systemic physiological interactions. Dysregulation of the gut microbiome is implicated in a wide spectrum of local and systemic diseases. Research has evolved from establishing associations to elucidating the mechanistic roles of gut microbes and developing targeted strategies for their modulation, with a growing emphasis on their bidirectional communication with other organ systems.

**Aim of review:**

This review aims to synthesize current knowledge on the composition and function of the gut microbiome, its functional crosstalk with the host, and its integral role in both health and disease. A major focus is placed on critically evaluating the mechanisms, efficacy, and applications of key microbiome-directed interventions—probiotics, prebiotics, synbiotics, and postbiotics—in maintaining or restoring gut-centric ecological balance.

**Key scientific concepts of review:**

The gut microbiome acts as a dynamic microbial organ essential for digestion, immune maturation, and metabolic homeostasis. Dysbiosis, characterized by a loss of beneficial microbes and an overgrowth of potential pathogens, is a critical factor in the pathogenesis of gastrointestinal disorders, metabolic diseases, and other systemic conditions. The gut microbiome engages in continuous bidirectional communication with distant organs, including the oral cavity, lungs, skin, and urinary tract, via specific axes (e.g., gut-oral, gut-lung, gut-skin), thereby exerting widespread influence on host physiology. Probiotics, prebiotics, synbiotics, and postbiotics represent complementary strategies to counteract dysbiosis and reestablish gut ecological integrity, ranging from introducing live beneficial bacteria to utilizing inactivated microbial cells and their bioactive metabolites. Enhancing the translational potential of these interventions requires deeper mechanistic insights and robust clinical validation.

## Highlights

• Elucidates gut microbiome composition and systemic organ interactions.
• Provides a critical comparative analysis of probiotics, prebiotics, synbiotics, and postbiotics.
• Evaluates emerging therapeutic strategies, including bacteriophages and live dietary microorganisms.
• Identifies key challenges and future directions for clinical translation.

## Graphical abstract

**Figure:**
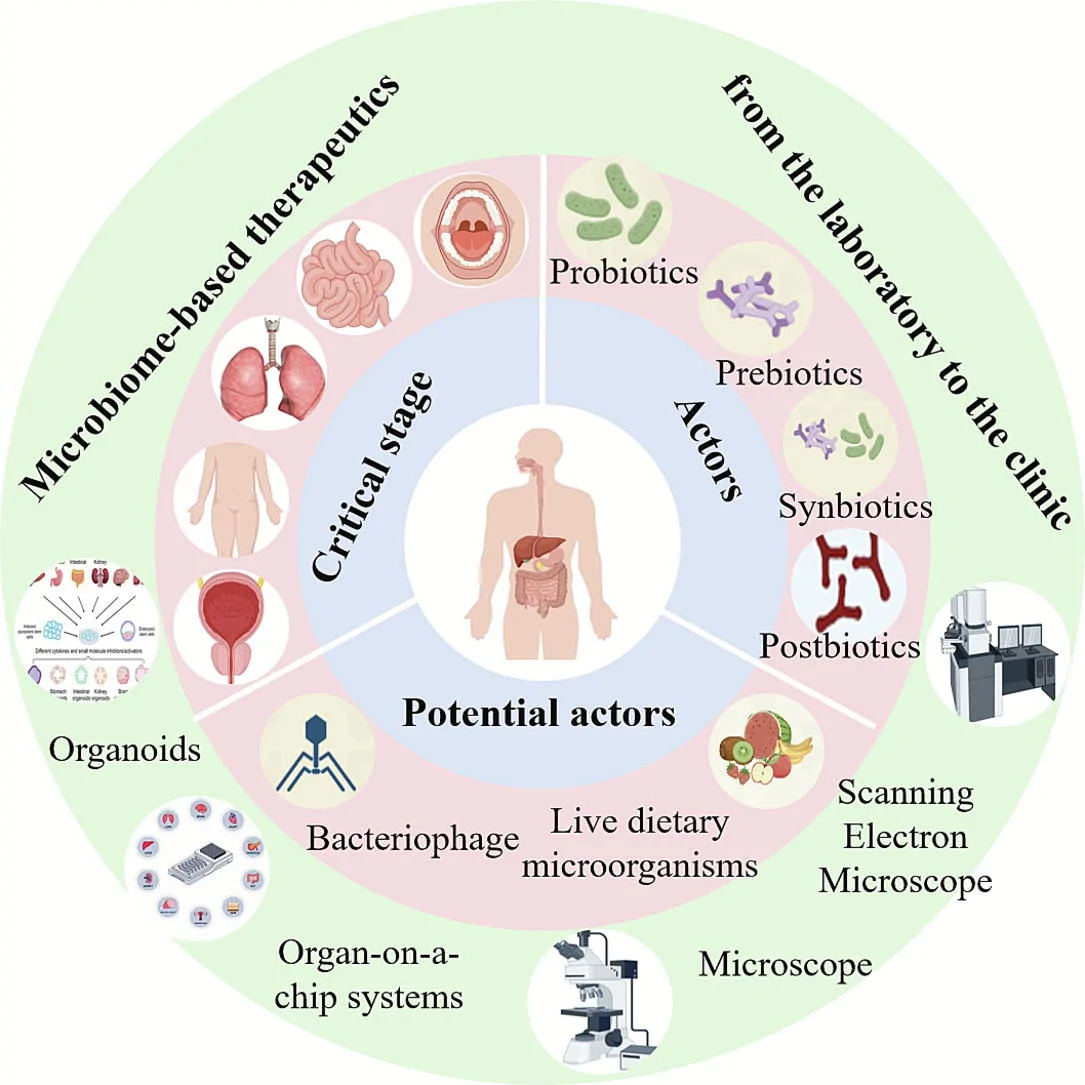

## Introduction

Over the past several decades, microbiome research has advanced at a rapid pace, emerging as a prominent focus within both the scientific community and public discourse [1]. The field originated in environmental microbiology and later shifted toward viewing eukaryotic organisms as inseparable from their microbial communities. This paradigm was greatly accelerated by culture-independent molecular surveys of the human gut [2], [3]. The human body harbors a sophisticated ecosystem in which trillions of microorganisms coexist with the host organism. In recent years, a holistic perspective rooted in holobiont theory has gained prominence, moving beyond the study of individual microbes in isolation [4]. Mutualistic interactions between the host and its microbiome contribute to maintaining health, whereas disease conditions are frequently associated with dysbiosis, an alteration in the composition of the microbial community. In this framework, pathogens represent only a small fraction of the total microbiota, and disease frequently arises when broader ecological changes allow opportunistic organisms to thrive [5]. Most microbial species play critical roles in ecosystem function and facilitate beneficial inter-species interactions that shape population dynamics and metabolic processes. Thus, the results of host-microbe interactions are influenced not just by the host or particular pathogens, but by the entire microbial ecosystem [6].

The term “microbiota” denotes all living microorganisms residing within this ecosystem, encompassing bacteria, archaea, fungi, algae, and small protists [7]. The concept of the microbiome has been further expanded to include not only these organisms but also the full range of molecules they produce, such as structural components, metabolites, and host-derived factors [2], [8]. While the inclusion of viruses, phages, and mobile genetic elements is still a matter of debate, they are being increasingly acknowledged as essential components of the microbial community [9]. Acting as a second genome, it engages the host in symbiotic relationships that range from beneficial to neutral to pathogenic, thereby profoundly influencing human health [10], [11].

The microbiome varies considerably across body sites and is highly individualized [12]. While a universal definition of a “healthy” microbiota is lacking, growing evidence shows that probiotics, prebiotics, and synbiotics can help maintain or restore a balanced microbial ecosystem, thereby supporting health [13]. More recently, postbiotics have been introduced as additional functional agents, collectively influencing the ecological dynamics of the microbiome to support symbiotic host–microbe relationships [14].

**Fig. 1:**
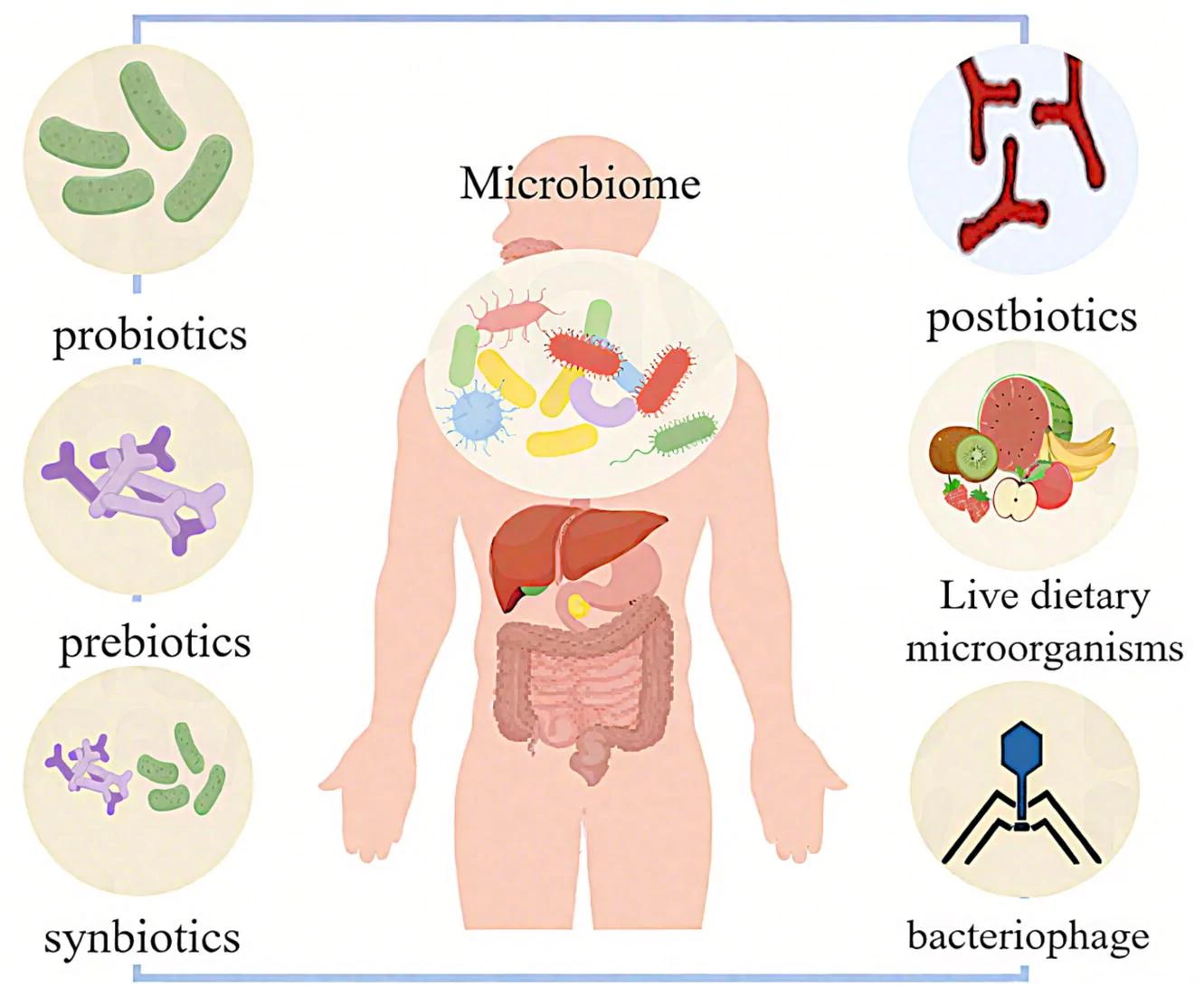
Intestinal interventions. Many interventions active in the intestinal theater can provide health benefits through oral (or rectal) administration.

The review will systematically delineate the unique compositional and functional attributes of these microbial communities, their implicated roles in a spectrum of diseases, and their intricate cross-talk with the host gut. A critical appraisal of the mechanistic underpinnings and clinical efficacy of established interventions—probiotics, prebiotics, synbiotics, and postbiotics—will be provided, alongside an exploration of novel approaches like bacteriophages and live dietary microorganisms (Fig. 1). By integrating contemporary research, this article aims to crystallize the paradigm shift from a gut-centric view to a holistic, systems-level understanding, thereby charting the course for future research and targeted therapeutic modulation of the human microbiome.

**Fig. 2:**
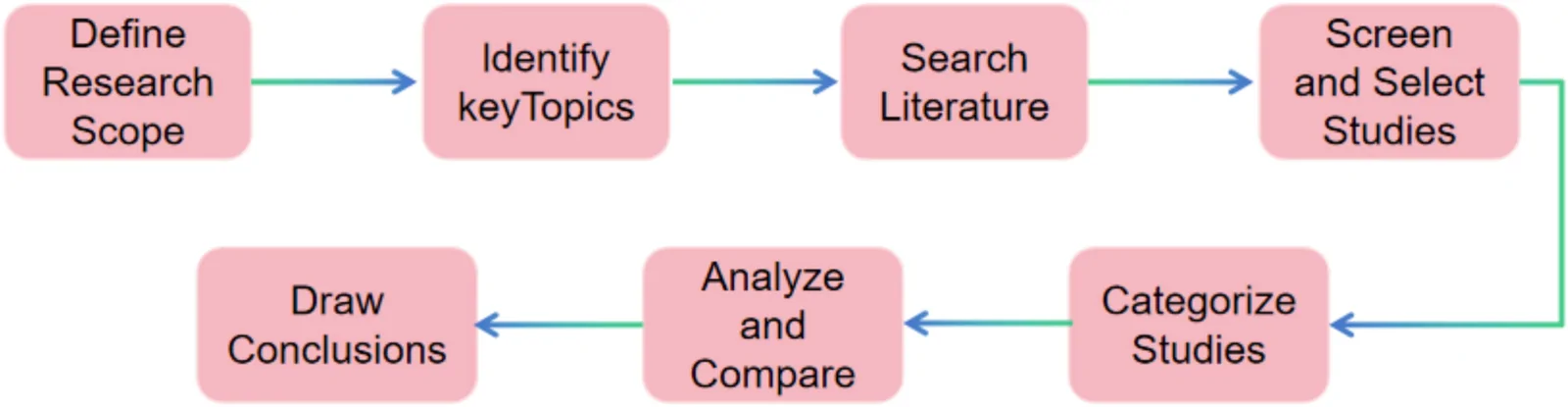
Overview of the review methodology.

The methodology adopted in this review is depicted in the flowchart presented in Fig. 2, which outlines the overall review workflow and offers a systematic illustration of key phases—ranging from defining the research scope to analyzing and synthesizing the collected findings. To achieve comprehensive literature coverage, electronic databases such as PubMed, Web of Science, and the Cochrane Library were systematically searched through a combination of subject headings and free-text keywords. The search timeframe extended from each database’s inception until November 2025, with restrictions applied to English-language publications. Furthermore, the reference lists of relevant review articles were manually examined to pinpoint studies that may have been overlooked. Literature screening and data extraction followed a double-blind, independent reviewer model. At the initial stage, two reviewers conducted independent screening of the titles and abstracts. Potentially eligible studies then underwent full-text retrieval and secondary screening. Any discrepancies that arose were resolved either through in-depth discussion or by adjudication from a third senior researcher. To assess the strength of evidence across various thematic domains, an evidence hierarchy was employed, ordered from highest to lowest rigor: systematic reviews and *meta*-analyses of randomized controlled trials (RCTs) involving human participants; individual well-designed human RCTs; *in vivo* animal studies; and *in vitro* mechanistic studies. Owing to substantial heterogeneity in study populations, intervention protocols, and outcome assessment measures, conducting a quantitative *meta*-analysis was not practicable. Instead, a narrative synthesis was employed to qualitatively summarize the evidence. Specific principles included categorizing studies according to predefined thematic domains and presenting study characteristics and results in a structured tabular format. After title/abstract screening and full-text assessment, a total of 252 studies were ultimately included in the review.

## The microbiome serves as a ‘critical stage’: A dynamic environment for host-microbiota interactions

### Definition and functional attributes of the microbiome

The microbiome constitutes the collective community of microorganisms along with their genetic material present in a given environment, including bacteria, fungi, viruses, and other microbes. These microorganisms assemble into consortia across diverse natural habitats—such as soil and aquatic systems—or host-associated niches like the human gut and respiratory tract, forming intricate multidimensional functional networks [15]. Illustrative of this complexity, the gut microbiome is frequently regarded as a “virtual organ” that profoundly influences host physiology through metabolite production and immune regulation [16].

A symbiotic relationship is typically maintained between the microbiome and its host, with its composition and functional profile being tightly linked to the specific environment [17]. As seen in sea buckthorn plants, root nodules are primarily colonized by *Frankia* species, which establish specialized symbiotic structures that enhance the host’s ability to thrive in nutrient-deficient conditions [18]. Similarly, in humans, the respiratory microbiome contributes to mucosal homeostasis by occupying available ecological niches and fine-tuning local immune responses; deviations from this balance are associated with heightened susceptibility to infections [19].

Far from static, the microbiome represents a dynamically regulated ecosystem influenced by host genetics and external factors, including diet and pharmaceutical agents. Patients diagnosed with coronary artery disease (CAD), for example, display markedly reduced gut microbial diversity accompanied by an increased abundance of *Bacteroidetes*, a profile correlated with dyslipidemia [20]. To reduce the complexity inherent in natural systems, synthetic microbiomes can be rationally designed, allowing precise manipulation of metabolic outputs and community behaviors [21].

The functional roles of the microbiome span metabolic regulation, immune modulation, and support of host evolutionary adaptation. In animal-associated ecosystems, microbial consortia are capable of synthesizing novel bioactive compounds—such as antimicrobial peptides and antioxidants—that mediate multi-organ effects via systemic communication axes like the gut-liver axis [22]. Likewise, in the realm of environmental bioremediation, engineered microbiomes are increasingly deployed to degrade pollutants or enhance nutrient cycling in soils, highlighting their broad applicability across biological scales [23].

### The gut microbiome and its interactions with other microbiomes

From birth onward, the human body is colonized by a diverse array of microorganisms, along with their genetic material and metabolic byproducts—collectively constituting the vertically transmissible human microbiome. This section spotlights several of the most functionally important microbial communities residing in the human body (Table 1) and provides a concise overview of the interactions between the gut and other organs.

**Table 1:** Primary microbiota colonising distinct anatomical sites and their associated disease links.

Microbiota	Microbiota count	PH	Predominant Microbes	Microbiota-associated Diseases	References
Gut Microbiota	10^7^–10^14^	5.7–8.5	Bacterial phyla: *Firmicutes* and *Bacteroides* Archaeal species: *Methanosphaera stadtmanae* and *Methanobrevibacter smithii*	Inflammatory bowel disease, irritable bowel syndrome, celiac disease, and colorectal cancer	[235], [236], [237]
Oral Microbiota	10^11^–10^12^	6.7–7.5	Bacterial phyla: *Actinobacteria,Bacteroidetes, Firmicutes, Fusobacteria, Proteobacteria*, and *Spirochaetes* Fungal genera: *Candida, Cladosporium*, *Saccharomycetales*, *Fusarium*, *Aspergillus*, and *Cryptococcus*	Dental caries, and periodontitis	[50], [57]
Respiratory Tract and Lung Microbiota	10^2^–10^9^	5.5–7.4	Bacterial phyla: *Firmicutes*, *Proteobacteria*, and *Bacteroidetes* Fungal species: *Candida albicans*, *Ceriporia lacerata*, *Saccharomycescerevisiae*, and *Penicillium brevicompactum* viruses: *Herpesviridae*	Asthma, and chronic obstructive pulmonary disease	[65], [73]
Skin Microbiota	10^11^	5.0–5.5	Bacterial phylum: *Actinobacteria*, *Firmicutes*, *Bacteroidetes*, and *Proteobacteria*	Atopic dermatitis	[76], [77]
Urinary Tract Microbiota	10^3^–10^4^	5.5–––6.5	Bacterial phylum: *Firmicutes*	Urgency urinary incontinence, and urinary tract infection	[87], [88], [91]

#### The human gut microbiome

The human microbiome is composed of a varied, ever-changing collection of microorganisms—including bacteria, fungi, and viruses—along with their genetic material and metabolic byproducts. These microbes establish themselves in the human body from the moment of birth and are passed down through generations vertically. The gut microbiota carries out a series of vital functions that help maintain the health of the host: it assists in breaking down complex carbohydrates, synthesizes essential vitamins, and participates in controlling the immune system. Furthermore, by vying with pathogenic microbes for nutrients and spatial resources, the gut microbiota prevents their colonization and potential invasion of the host. It also produces a wide variety of bioactive compounds—short-chain fatty acids (SCFAs), for example—that have anti-inflammatory effects and strengthen the gut barrier’s integrity. Factors like diet, antibiotic usage, and other environmental signals can significantly upset the balance and composition of the gut microbiota, making it a key mediator in the host’s defensive mechanisms [24].

Inflammatory bowel disease (IBD) encompasses a group of heterogeneous chronic inflammatory disorders, with two primary distinct conditions: Ulcerative colitis (UC) and Crohn's disease (CD) [25], [26]. Although the exact triggers for the initial inflammatory episode and subsequent relapses remain unclear, current evidence indicates that in genetically susceptible individuals, an immune response against intestinal microbial antigens under specific environmental conditions can lead to inflammatory episodes [27]. Countless IBD-related susceptibility genes are linked to the host’s reactions to gut bacteria, suggesting that the gut microbiota also contributes to the development of IBD [28]. Dysbiosis—characterized by decreased microbial diversity, loss of beneficial bacterial taxa, and/or excessive growth of harmful bacteria—might have a role in the onset of IBD [29]. The most notable changes in the gut microbiota of patients with IBD include a drop in bacterial diversity, together with lowered levels of *Bacteroidetes* and *Firmicutes*, and a simultaneous increase in the number of *Proteobacteria* [30]. In IBD, the impaired mucus layer allows luminal bacteria to penetrate and invade the submucosa, thus triggering proliferative and inflammatory responses. Subsequent mucosal damage from inflammation further exposes the underlying tissue to more bacteria, creating a self-perpetuating cycle of antigen exposure and mucosal injury [31].

Colorectal cancer (CRC) is the third most prevalent malignancy worldwide, with a growing occurrence rate in people younger than 50, a trend associated with particular dietary components and eating habits that impact the gut microbiota [32]. When compared to people in good health, those with CRC show reduced bacterial variety and population size, along with a higher proportion of *Firmicutes* and *Bacteroidetes* [33]. Several specific bacterial species have been associated with the start and advancement of CRC, such as *Fusobacterium nucleatum*, *Escherichia coli*, *Enterococcus faecalis*, *Streptococcus gallolyticus*, and enterotoxigenic *Bacteroides fragilis* [34]. A multitude of evidence highlights the tumor-promoting impact of *Streptococcus gallolyticus* on colon cells. When colon cells are cultured with this bacterium, they display elevated levels of β-catenin, c-Myc, and proliferating cell nuclear antigen (PCNA)—all of which are transcription factors linked to cancer progression. In mouse models, the administration of *S. gallolyticus* resulted in a larger quantity of tumors, a heavier tumor load, more serious dysplasia, and enhanced cell proliferation, as well as increased β-catenin staining in colonic crypts, when contrasted with control groups [35]. Additionally, CRC patients show considerably higher levels of this bacterium in comparison to healthy individuals. It is also suggested that CRC-specific changes—such as increased bile acid concentrations—may promote the colonization of *S. gallolyticus*, creating a self-sustaining cycle that maintains high abundances of this bacterium in the gut [36].

In recent years, the participation of the microbiome in obesity and related metabolic disorders—including type 2 diabete (T2D) [37], and non-alcoholic fatty liver disease (NAFLD) [38]—has been thoroughly investigated. The gut bacterial community plays a crucial role in governing multiple facets of metabolic disorders, mainly by generating a diverse array of metabolites—from SCFAs [39] to structural large molecules like peptidoglycan and lipopolysaccharide (LPS) [40]. These metabolites interact with host receptors, either activating or inhibiting signaling pathways that may support or compromise host health.

A century ago, researchers discovered viruses capable of killing bacteria—known as bacteriophages—in the feces of World War I soldiers. Today, these viruses are gaining renewed attention for their potential role within the human body. Bacteriophages are widely present in various environments, including oceans and soil. Recent studies indicate that the human body can absorb up to 30 billion bacteriophages daily through the gut [41]. Furthermore, studies indicate substantial quantities of prephages reside within the human gut, extensively integrated into the genomes of gut bacteria [42]. Although decades of antibiotic use overshadowed phages as therapeutic agents, the emergence of multidrug-resistant bacteria and deepening understanding of gut microbiota's role in health and disease have brought phages back into the research spotlight [43]. Phages not only serve as potent tools for preventing and treating pathogenic infections but also enable precise editing of the gut microbiota, demonstrating promising therapeutic potential for various gastrointestinal disorders [44]. Although multiple therapeutic approaches have been proposed, this field still requires further basic and preclinical research, as well as rigorously designed randomized, double-blind, placebo-controlled trials to advance its development.

The human microbiome shows remarkable diversity, with notable variations seen among different people. Research indicates that the human gut microbiome might comprise hundreds to thousands of distinct bacterial species, and there are also substantial differences at higher taxonomic levels [45]. Nevertheless, despite this high degree of diversity, certain core microbial species are found in most of the population—a sign that a group of basic functions crucial for human health exists [24].

The composition of the gut microbiota is generally stable and can recover to its original state after short-term disruptions, thanks to its self-regulatory capacity. However, prolonged exposure to stress factors that are widespread in modern lifestyles—such as Western diets, food additives, environmental pollutants, and frequent antibiotic use—can induce chronic changes in the gut microbiota. These persistent modifications may promote the proliferation of pathogenic microorganisms, which might lead to adverse health consequences for the host [46].

The makeup of the gut microbiota is molded by a combination of host-specific and external environmental elements, with significant effects on human well-being. Host determinants include age, delivery mode, liver metabolic capability, immune function, the generation of specific bioactive lipids, and overall physiological condition. Genetic variation further impacts microbial diversity, resilience, and an individual’s vulnerability to disease as well as response to therapies. At the same time, external influences—including diet, hygiene, exposure to pollutants and chemicals, psychological stress, and lifestyle elements like physical activity, sleep patterns, and social interactions—also play a vital part in shaping the microbial community [47].

Over the past two decades, research into the gut microbiota has evolved from clinical observations to mechanistic investigations; however, many studies continue to conflate correlation with causation. Establishing definitive causal relationships is now crucial for the development of effective microbiota-targeted or metabolite-based therapeutic interventions.

#### The human oral microbiome and its interaction with the gut microbiome

The gut-oral axis denotes the bidirectional communication between the microbial communities inhabiting the gut and those in the oral cavity. The gut microbiome, composed of trillions of microorganisms, is crucial for overall health, as it aids in regulating immunity, metabolism, and inflammatory responses. Likewise, the oral microbiome is vital for oral health by defending against pathogens and regulating local immune function. These two ecosystems are linked through the bloodstream and shared immune pathways, enabling the exchange of microbial metabolites, immune cells, and inflammatory signals [48].

The human oral cavity is frequently characterized as the body’s “second genomic repository,” exhibiting microbial complexity surpassed only by that of the gut. This site is inhabited by a diverse array of microorganisms—including bacteria, fungi, viruses, and protozoa—that occupy two major ecological niches. One niche encompasses the hard surfaces, such as tooth enamel and dental prostheses, while the other consists of soft tissues like the dorsal tongue, buccal mucosa, and gingival sulcus [49].

It has been suggested that oral microbes can gain entry into the circulatory system during mastication, as well as during routine oral hygiene practices like tooth brushing and flossing, and then travel to distant locations throughout the body [50]. In this complex setting, interactions between pathogenic and commensal microorganisms and the host immune system are critically important. Notably, oral squamous cell carcinoma (OSCC) and certain gastrointestinal (GI) cancers—including CRC and pancreatic ductal adenocarcinoma (PDAC)—have been intriguingly associated with the oral microbiome [51]. Studies suggest that *Porphyromonas gingivalis* contributes to tumor development by interfering with cellular signaling pathways. This bacterium secretes nucleoside diphosphate kinase (NDK), which degrades extracellular adenosine triphosphate (ATP) and thereby inhibits ATP-mediated activation of the purinergic receptor P2X7 on macrophages. As a result, inflammasome formation and interleukin (IL)-1β secretion are suppressed, creating conditions that favor tumorigenesis [52]. Similarly, infection with *Fusobacterium nucleatum* has been shown to upregulate matrix metalloproteinase-13 (MMP-13), promoting cell migration through activation of Etk/BMX, p70 S6 kinase (p70^S6K^), and RhoA kinase. Furthermore, *F. nucleatum* stimulates p38 mitogen-activated protein kinase, leading to heat shock protein-27 (HSP-27) activation and subsequent induction of matrix metalloproteinase-9 (MMP-9) and MMP-13—key enzymes involved in tumor invasion and metastasis [53]. In contrast, commensal bacteria such as *Streptococcus salivarius strain* K12 exert protective effects by producing bacteriocins that inhibit colonization by Gram-negative pathogens and help sustain immune homeostasis [54]. Similarly, chronic gut dysbiosis also contributes to systemic inflammation, marked by increased levels of circulating proinflammatory cytokines and immune cells. These mediators can alter the oral microenvironment, promoting carcinogenesis. Furthermore, gut dysbiosis hinders the differentiation and functional performance of immune cells—including T cells, macrophages, and dendritic cells—thereby compromising immune surveillance within the oral cavity [55].

At the systemic level, dysbiosis of the oral microbiome has been strongly associated with a range of chronic diseases. Periodontal disease causes microbiome dysbiosis and increases the load of *viridans streptococci*. This, consequently, enables bacteria to enter the bloodstream through a leaky endothelium—an entry pathway also enabled by periodontal procedures like scaling, tooth brushing, and tooth extractions. Repeated exposure of the cardiac valve endothelium to viridans streptococci leads to valve thickening, platelet activation, fibrin deposition, and the development of sterile lesions known as thrombotic endocarditis [56]. Moreover, in osteoporosis, the primary evidence centres on alveolar bone homeostasis, with molecular evidence linked to the oral microbiome having been identified. [57]. The oral commensal microbiome helps maintain alveolar bone homeostasis by modulating osteocalcin expression in osteoblasts through specific transcription factors. Osteocalcin, in this context, acts to curb excessive bone mineralization while promoting the function of both osteoblasts and osteoclasts [58]. Given the close physical proximity of the oral microbiome and alveolar bone, it is believed to be a key regulator of bone homeostasis in this region [59]. Nonetheless, the exact molecular pathways by which these commensals exert their regulatory impacts remain unclear [60]. Furthermore, the oral microbiome is increasingly implicated in non-communicable chronic diseases (NCDs); both epidemiological and mechanistic studies suggest its involvement in the onset and advancement of obesity, T2D, malignancies, as well as various neuropsychiatric disorders (NPDs) [61]. Consequently, the oral microbiome is increasingly regarded as a legible health record.

Parallel to advancements in gut metagenomics, integrative multi-omics approaches—encompassing metagenomic, metatranscriptomic, metabolomic, and epigenomic data—are being employed to develop systems biology models of host-microbe interactions. These approaches hold promise for early risk prediction, identification of therapeutic targets, and personalized microbiome restoration. Future clinical applications may include probiotic or postbiotic formulations, targeted phage therapy, and artificial intelligence-guided microbiome reprogramming, potentially transforming oral microbiome modulation into a clinical reality.

#### The human respiratory tract and lung microbiomes and its interaction with the gut microbiome

A bidirectional interplay exists between the gut microbiota and lung ailments. Respiratory infections and pulmonary disorders are capable of diminishing gut microbial diversity, thereby triggering dysbiosis [62]. On the flip side, a perturbed gut microbial community can also impact the advancement and clinical results of pulmonary conditions.

For decades, the respiratory tract was presumed to be sterile, largely owing to the repeated failure of conventional culture methods to isolate viable bacteria from airway specimens. It is now recognized that inhaled environmental microbes initially colonize the upper airway (pharynx and larynx) and may subsequently descend—via micro-aspiration—into the lower airway (trachea and bronchi) through either oral or nasal pathways [63]. Epidemiological associations have been established between these transient or resident microbial communities and both the frequency and severity of asthma attacks as well as exacerbations of chronic obstructive pulmonary disease (COPD). Nonetheless, the airway microbiome remains inadequately characterized, underscoring the urgent need for large-scale longitudinal studies [64].

The human lung, once considered sterile, is now known to harbor a dynamic microbial consortium referred to as the lung microbiome. Perturbations of this community have been documented across a wide array of pulmonary disorders, including chronic diseases such as COPD, asthma, and bronchiectasis; acute conditions like bacterial and viral pneumonia, sepsis-associated acute respiratory distress syndrome (ARDS), and Corona Virus Disease 2019 (COVID-19); as well as specialized clinical scenarios including post-transplant immunosuppression, lung cancer, and human immunodeficiency virus (HIV)-associated pulmonary disease [65]. Mounting evidence suggests that these microbial changes regulate not only local pulmonary immune function but also systemic inflammatory reactions. However, the precise molecular mechanisms—through which specific microbial taxa produce structural ligands that interact with host pattern-recognition receptors, transcription factors, or epigenetic regulators—remain largely elusive.

Emerging evidence indicates the GI system’s involvement in the origin, development, and outcome of pneumonia. GI symptoms—such as diarrhea—are seen in 2–20 % of COVID-19 cases [66], and are associated with impaired tryptophan synthesis [67]. Furthermore, studies in murine models demonstrate that infections with the influenza virus or respiratory syncytial virus, as well as experimental acute lung injury, can induce gut dysbiosis. In the lung injury model, bacteria were found to translocate from the lungs to the intestines via the bloodstream, elevating the intestinal microbial load and disrupting commensal diversity [68]. These findings highlight the function of the gut-lung axis, wherein pulmonary infections may alter gut microbiota and trigger GI symptoms through immune regulation. Despite this, it remains controversial whether gut dysbiosis is merely a result of pneumonia.

Gut microbiota dysbiosis can adversely affect the lungs by altering the pulmonary microbiota, worsening inflammation, and serving as a poor prognostic factor in respiratory infections. For instance, severe acute respiratory syndrome Coronavirus 2 (SARS-CoV-2) replicates in the gut and may disseminate to the lungs by modulating inflammatory responses and angiotensin-converting enzyme 2 (ACE2) expression [69]. Intriguingly, tryptophan has been shown to upregulate ACE2 and broad-spectrum neutral amino acid transporter (B0AT1), activating the mammalian target of rapamycin (mTOR) pathway to aid intestinal repair [70]. Specific gut microbes, such as *Enterobacteriaceae*, are linked to community-acquired pneumonia (CAP), while overall flora imbalance may produce autoinducer-2 (AI-2) to disrupt brain-gut signaling and exacerbate post-stroke pneumonia [71]. Conversely, beneficial gut microbes and their metabolites offer protection; for example, restoring a healthy gut microbiota can eliminate *Pseudomonas aeruginosa* colonization, thereby preventing its infection and spread [72].

Research progress is impeded by several unique challenges. Biologically, the lung microenvironment is characterized by low microbial biomass, high levels of host DNA background, variable oxygen tensions, and continuous mucociliary clearance. Technically, sample acquisition is inherently invasive, and even minimal oropharyngeal contamination can compromise the fidelity of lung-specific microbial profiling. Therefore, the development of specialized experimental workflows—such as ultra-low-biomass DNA extraction kits, rigorous negative controls, and integrated bioinformatic decontamination algorithms—as well as advanced analytical frameworks (e.g., microbial source-tracking models and multi-kingdom reference databases) is essential before the lung microbiome can be reliably translated into mechanistic understanding or therapeutic applications [73].

#### The human skin microbiome and its interaction with the gut microbiome

Research is increasingly demonstrating that there is notable signaling and cellular crosstalk between the seemingly unrelated gut and skin connection referred to as the gut-skin axis. Interaction between these two organs is facilitated by a variety of cytokines, microbial metabolites, hormones, and neurotransmitters, all of which play crucial parts in preserving tissue homeostasis and coordinating systemic responses [74], [75].

As the body’s largest and most environmentally exposed organ, human skin harbors a remarkably stable microbiota despite continuous exposure to transient microorganisms. It serves not only as a physical barrier against pathogen invasion but also as a niche for resident commensals, including both bacteria and fungi [76]. The cutaneous microbial community is dominated by three bacterial phyla—*Actinobacteria, Firmicutes, and Proteobacteria*—with key representatives such as the commensals *Corynebacterium tuberculostearicum, Cutibacterium acnes, and Staphylococcus epidermidis*. In healthy adults, sebum-rich regions are predominantly colonized by lipophilic *Cutibacterium* species, whereas *Staphylococcus* and *Corynebacterium* species thrive in humid and moist areas, including the armpits, elbow bends, and feet [77].

Atopic dermatitis (AD) is a polygenic and phenotypically heterogeneous disorder underpinned by three interconnected mechanisms: a compromised epidermal barrier, a chronically dysregulated immune response, and a profoundly reprogrammed skin microbiome. Both metagenomic and culture-based studies consistently demonstrate that disease flares coincide with a loss of cutaneous biodiversity and the marked expansion of *Staphylococcus aureus*. Notably, *S. aureus* genotypes isolated from AD lesions are not passive colonizers; they are genetically and functionally distinct from commensal strains found in healthy individuals. Comparative genomic analyses have identified divergent loci encoding virulence factors, adhesins, and pro-inflammatory molecules. These strain-specific attributes facilitate adhesion to compromised keratinocytes, secretion of pore-forming toxins that exacerbate corneocyte damage, and initiation of immune cascades. The result is a self-reinforcing cycle in which microbiome disruption, barrier impairment, and immune dysregulation collectively drive AD pathogenesis [78].

Resident skin microbes typically cooperate to prevent pathogenic colonization; however, under dysbiotic conditions, commensals can transition into a pathobiont state. Acne vulgaris—an inflammatory condition of the pilosebaceous units—serves as a representative example: Cutibacterium acnes, a dominant commensal in post-adolescent sebaceous skin, becomes pathogenic when ecological equilibrium is disrupted [79]. This common dermatosis arises from complex interactions between host skin physiology and the cutaneous microbiome [80].

The prevailing view of the gut-skin axis posits a primarily unidirectional communication, where the gut influences the skin. This perspective is rooted in the gut microbiota's central role in systemic metabolic and immune functions. Gut microbes convert indigestible polysaccharides into essential nutrients like vitamin K and B vitamins, which support wound healing and processes such as DNA replication and methylation, respectively [81]. Additionally, bacterial metabolites, notably SCFAs, function as key signaling molecules in the gut-skin axis by mitigating skin inflammation [82].

In contrast to the extensively investigated influence of diet and the gut microbiome on skin health, the reverse pathway of the gut-skin axis remains less explored. While external factors like pollution, smoking, and sun exposure directly affect the skin [83], the latter also plays a role in systemic signaling. Vitamin D (calciferol), a fat-soluble vitamin obtained from dietary sources like oily fish, eggs, and fortified foods, is also synthesized in the skin upon ultraviolet radiation B (UVB) exposure from 7-dehydrocholesterol [84]. Recent research shows that UVB exposure on the skin can alter the gut microbiota in women with vitamin D insufficiency [85]. The underlying mechanisms, though not fully clear, may involve enhanced innate immunity, reduced inflammation, and improved intestinal barrier function [86].

The microbial communities on human skin constitute a dynamic ecosystem that is essential for cutaneous health and tissue homeostasis. Future studies are therefore essential to further delineate their composition and function—particularly across diverse populations, to monitor microbiome changes associated with the onset of dermatologic diseases, and to advance their potential for therapeutic manipulation.

#### The human urinary tract microbiome and its interaction with the gut microbiome

The gut microbiome acts as a vital regulator of systemic physiology, including susceptibility to infectious ailments. While this habitat serves as a storage site for uropathogenic *Escherichia coli*, understanding of its role in urinary tract infections (UTIs) remains inadequate.

For decades, the urinary bladder was conventionally regarded as a sterile environment; any bacteria detected within it were automatically classified as pathogens. This dogma has been overturned by advances in sampling techniques and the application of culture-independent sequencing methods. It is now established that a resident urinary microbiota—termed the “urobiome”—exists and contributes to urological health rather than representing contamination [87]. This low-biomass community engages in sophisticated dialogue with the urothelium and the underlying mucosa-associated lymphoid tissue (MALT). Through quorum-sensing mechanisms and metabolite exchange, commensal microbes help regulate epithelial barrier integrity, modulate innate immune responses, and occupy ecological niches that might otherwise be exploited by uropathogens [88]. Disruption of this equilibrium—due to antibiotic use, estrogen depletion, or catheterization—can lead to dysbiosis, increasing susceptibility to UTIs and potentially exacerbating symptoms such as urgency, incontinence, and chronic pelvic pain [89].

Anatomic factors strongly influence microbial colonization patterns. In females, the short distance between the vaginal introitus and urethral meatus facilitates continuous bidirectional microbial exchange. As a result, the vaginal microbiota frequently serves as a primary source for bladder communities [90]. Clinical observations corroborate this relationship: recurrent UTIs are reduced following vaginal instillation of Lactobacillus crispatus, which promotes a lactobacilli-dominated environment, whereas bacterial vaginosis—characterized by dominance of anaerobes such as Gardnerella vaginalis—is associated with significantly higher UTI incidence [91]. Recent research has confirmed the dynamic nature of the urinary microbiome across the lifespan. In females, studies that identified distinct microbiomes in prepubescent, reproductive-age, and postmenopausal individuals have indicated that sex hormones may act as potential modulators of their composition, while also pinpointing prevalent species that could signal dysbiosis. In male children, research shows the presence of a cultivable urinary microbiota that changes with age or urologic history, but not with the mode of delivery [92].

Diagnostic strategies based on metabolites hold promise for addressing the time-consuming nature of traditional urine cultures. For instance, detecting specific microbial metabolites in urine, such as guanidine and N6-methyladenine, can rapidly distinguish between different types of pathogen infections, thereby reducing antibiotic overuse [93]. Future spatial metabolomics technologies may enable direct visualization of the precise locations where these metabolites are produced within bladder or urethral tissues.

*Uropathogenic Escherichia coli* (UPEC) is the leading cause of UTIs, though other bacterial and fungal pathogens also contribute substantially to clinical cases. The gut serves as the primary reservoir for uropathogens such as UPEC, providing a staging ground from which they can access and ascend the genitourinary tract to infect the bladder [94]. Although these bacteria can form persistent reservoirs within the bladder and vaginal epithelium, the gut microbiota remains their principal source [95]. Due to the distinct physiological and chemical conditions of the gut and urinary tract, studies have sought to identify UPEC-specific fitness factors that confer uro-virulence. Notably, while all *E. coli* carry the fim operon for type 1 fimbriae (pili) assembly, unique mutations in the FimH adhesin gene of UPEC strains enhance binding to mannosylated receptors on bladder epithelial cells. A key unresolved question is whether commensal gut *E. coli* acquire pathogenic traits within the gut, or if a pre-adapted UPEC subpopulation already resides in the gut and is selected upon entering the bladder niche [96].

However, it is important to note that when collecting urine for microbiome testing, in addition to physiological differences such as gender and age, one must also consider that, despite providing subjects with detailed collection instructions, urethral contamination may occur during the collection process, potentially affecting the results [97].

Future efforts involving longitudinal multi-omics studies—integrating host transcriptomics, metabolomics, and spatial microbiome mapping—are expected to elucidate how specific microbial taxa and their metabolites influence epithelial signaling and neutrophil recruitment. These mechanistic insights could enable the development of precision diagnostics, such as metagenomic urine profiles predictive of recurrence, and non-antibiotic therapies including live biotherapeutics, bacteriophage preparations, and quorum-sensing inhibitors. By diminishing reliance on broad-spectrum antibiotics, urobiome-centered medicine represents a promising avenue for reducing antimicrobial resistance while promoting urinary tract health.

### Summary

This section outlines the diverse microbial communities inhabiting various parts of the human body and their interactions with the gut microbiome. These communities are closely linked to host physiology and have coevolved. They exert profound effects on both health maintenance and disease progression, making the microbiome a highly promising therapeutic target.

## Probiotics, prebiotics, synbiotics, and postbiotics as ‘actors’: active remodelling of gut microbiome functions

Trillions of microbial inhabitants in the gut play a crucial role in regulating digestion, immune function, and even mental health; maintenance of this complex ecosystem is essential, as its disruption has been linked to metabolic disorders. This section describes four functional food agents—prebiotics, probiotics, synbiotics, and postbiotics—whose dietary intake has been shown to beneficially modulate the gut microbial community (Table 2).

**Table 2:** Health effects mediated by probiotics, prebiotics, synbiotics, and postbiotics: an overview.

Category	Definition	Exemplars	Mechanisms of Action	Evidence tier	References
Probiotic	Live microorganisms, when applied in sufficient quantities, can bring health benefits to the host	*Lactobacillus plantarum*, *Lactobacillus reuteri*, and *Lactobacillus acidophilus*	Enhance gut barrier function, produce antimicrobial substances, modulate immune responses	Human randomized controlled trial, and animal experimentation	[108], [110], [116], [219]
Prebiotic	Substrates selectively utilized by the host microbiota that bring health benefits to the host	Xylo-oligosaccharides, galacto-oligosaccharides, and fructo-oligosaccharides	Promote the growth of beneficial bacteria and alter gut microbiota composition	Human randomized controlled trial, and animal experimentation	[125], [130], [132], [140]
Synbiotic	Mixtures comprising live microorganisms and substrate(s) selectively utilized by host microorganisms that confers a health benefit on the host	*Bifidobacterium lactis* HN019*, Lactobacillus rhamnosus* HN001*,* and Fructooligosaccharide *Lactobacillus suilingensis* AF91-01CMCA*,and* Inulin	Improve gut microbiota diversity, enhance the absorption of nutrients, synergistic effects between probiotics and prebiotics	Human randomized controlled trial, and animal experimentation	[15], [137], [139], [143]
Postbiotic	Probiotic microbial preparations and/or their components that confer health benefits to the host	Lipopolysaccharides,SCFA, and Trimethylamine Oxide	Influence gut microbiota composition, reduce intestinal inflammation, enhance antioxidant enzyme activity	Human randomized controlled trial, and animal experimentation	[159], [168], [174], [186]

### Probiotic

Per the definition jointly issued by the Food and Agriculture Organization of the United Nations (FAO) and the World Health Organization (WHO), probiotics refer to “live microorganisms which, when administered in adequate amounts, confer a health benefit on the host.” [98]. Present in both supplemental formulations and fermented foods, these microorganisms transiently or permanently colonize the intestinal tract, modulating microbial equilibrium and interacting with immune cells to enhance host defense mechanisms [99], [100]. Their preventive potential against a range of disorders supports their growing use as accessible therapeutic adjuncts; however, the precise signaling pathways through which they mediate immunomodulatory effects remain to be fully elucidated [101].

The vast majority of identified probiotics are Gram-positive organisms, with *Lactobacilli* and *Bifidobacteria* representing the most prominent and historically central genera [102]. Beyond these well-characterized taxa, the scope of probiotic agents includes an eclectic array of microbes: avirulent strains of *Escherichia coli* that produce microcins, bacteriocin-producing *Enterococcus* and *Pediococcus* species, and the yeast *Saccharomyces boulardii*, which has been demonstrated to enhance intestinal barrier integrity and suppress inflammatory pathways [103]. Further expanding this concept, emerging evidence indicates that certain commensal streptococci—particularly *Streptococcus oralis* and *Streptococcus salivarius*—can translocate from the oral cavity to the gut, where they promote a tolerogenic immune environment and suppress pathobiont expansion, thereby broadening the definition of health-promoting microbes [104].

Irritable bowel syndrome (IBS) is a common functional GI disorder, featuring chronic abdominal pain along with changes in bowel habits—without any detectable structural or biochemical abnormalities [105]. Its pathogenesis is multifactorial, involving a range of disturbances such as abnormal gut motility, visceral hypersensitivity, altered immune function, increased intestinal permeability, gut microbiota dysbiosis, and dysregulated gut-brain axis communication [106]. Growing evidence suggests that intestinal dysbiosis may be a central factor underlying many of these pathological features [107]. Consequently, modulating the microbiota composition is increasingly considered a valuable therapeutic approach for IBS. In this context, probiotic supplementation has emerged as a promising strategy. A randomized, double-blind, placebo-controlled trial demonstrated that a multi-strain probiotic containing *Lactobacillus*, *Bifidobacterium*, and *Streptococcus thermophilus* was well-tolerated and safe in patients with diarrhea-predominant IBS (IBS-D). The treatment led to significant improvements in overall IBS symptoms, as well as in pain severity and quality of life, indicating beneficial effects for adult IBS-D patients [108].

Among the numerous functions attributed to probiotics, their ability to fine-tune immune responses is considered one of the most clinically significant. This immunomodulatory capacity is mediated through dynamic molecular interactions involving multiple cell types along the mucosal interface, including dendritic cells, macrophages, monocytes, lymphocytes, and intestinal epithelial cells [109]. These interactions lead to measurable enhancements in host defense, such as increased luminal lysozyme secretion, IL-8-mediated neutrophil recruitment, modulated transforming growth factor-β (TGF-β) signaling that limits fibrosis, and elevated immunoglobulin (Ig) M levels in both systemic and mucosal compartments. Additionally, peripheral blood lymphocytes exhibit enhanced phagocytic activity, reflecting the systemic immunologic impact of local probiotic administration [110]. The immunomodulatory and ecological effects of probiotics are illustrated by *Lactobacillus rhamnosus* HDB1258 (HDB1258). Oral administration in C57BL/6 mice enhanced innate immune function—evidenced by increased natural killer (NK) cell cytotoxicity—and induced favorable shifts in gut microbiota composition. Metagenomic analyses revealed an expansion of beneficial taxa, including *Lactococcus* and *Sutterella*, alongside a reduction in opportunistic pathogens [111]. These findings identify HDB1258 as a promising immunobiotic candidate capable of simultaneously modulating immune function and microbial ecology to support host health.

Probiotic supplementation has been proposed as an adjunctive strategy in the management of type 2 diabetes mellitus (T2DM), with potential benefits including improved lipid profiles and glycemic control, likely mediated through immunoregulatory mechanisms [112]. Experimental studies indicate that multi-strain *Lactobacillaceae* formulations can reduce fasting blood glucose, attenuate systemic inflammation, and mitigate tissue damage in the liver, kidneys, and pancreas. These effects are associated with activation of the PI3K/AKT pathway and increased production of SCFAs. Specific strains such as *Lactobacillus plantarum* 22*, Lactobacillus plantarum* 25*, Lactobacillus fermentum* 11, and *Lactobacillus fermentum* 305 show particular promise for T2DM management [113].

Probiotics are increasingly recognized as practical interventions for metabolic disorders. Evidence indicates that specific strains, when administered at adequate doses, can significantly reduce serum cholesterol levels. *Lactobacillus reuteri* (*L. reuteri*), a well-studied commensal with sustained gut colonization capacity, demonstrates hypocholesterolemic and systemic anti-inflammatory properties. Beyond lipid modulation, *L. reuteri* and its metabolite gamma-aminobutyric acid (GABA) have been reported to ameliorate myocardial injury following ischemia/reperfusion surgery [114]. Similarly, *Lactobacillus fermentum* MJM60397 has been evaluated for safety and functionality, demonstrating antibiotic susceptibility, no mucin degradation, absence of hemolytic activity, and no production of biogenic amines. This strain also exhibits high gastric acid and bile salt tolerance, adherence to intestinal epithelial cells, and bile salt hydrolase activity—properties that collectively promote bile acid deconjugation, fecal excretion, and consequent cholesterol reduction [115]. *Lactiplantibacillus pentosus* KS6I1 has also been shown to reduce serum cholesterol in diet-induced hypercholesterolemic mice, linked to enhanced fecal excretion of bile acids and cholesterol, supporting its potential as a probiotic for hypercholesterolemia management [116].

The link between obesity and the gut microbiota is well-documented, and certain probiotics can mitigate diet-induced weight gain. For instance, the human symbiont *Dysosmobacter welbionis* attenuates metabolic stress by reducing fat accumulation and modulating brown adipose tissue (BAT) thermogenesis under high-calorie conditions [117]. Similarly, *Lactobacillus acidophilus* supplementation reduces body weight, adiposity, systemic inflammation, and insulin resistance in high-fat diet (HFD)-fed animals. Mechanistically, *L. acidophilus* enhances BAT activation, normalizes energy expenditure, improves glucose and lipid metabolism, and reverses diet-induced dysbiosis—reducing the *Firmicutes/Bacteroidetes* ratio and abundance of endotoxin-producing Gram-negative bacteria. It also reinforces intestinal barrier function, thereby reducing metabolic endotoxemia and suppressing TLR4/NF-κB signaling, which collectively ameliorate dyslipidemia, NAFLD, and insulin resistance [118]. These animal study findings suggest that L. acidophilus may offer potential benefits for human-related diseases.

However, it is crucial to note that while the aforementioned mechanistic evidence from animal studies is encouraging, extreme caution is warranted when directly extrapolating these findings to human health outcomes. It is noteworthy that humans and rodents exhibit significant physiological differences. Currently, high-quality clinical trials directly validating the efficacy of *Lactobacillus* species in improving myocardial injury, or the therapeutic effects of *L. acidophilus* on human hyperlipidemia and NAFLD, remain scarce. Therefore, these positive animal study findings primarily provide insights into the mechanism of action of probiotics and lay the theoretical foundation for future targeted clinical trials in humans. Until more conclusive human evidence is obtained, any conclusions regarding its efficacy for human diseases should be considered preliminary and speculative.

Although the benefits of probiotics are well-established and widely recognized, it is important to acknowledge that adverse effects, while exceptionally rare, may still occur [119]. Didari et al. concluded that probiotics are safe for healthy individuals, but may pose potential risks for severely ill, hospitalized, postoperative, or immunocompromised patients [120]. Furthermore, although occurring very infrequently, probiotic strains have been documented to cause infections in humans. For example, Naqvi and colleagues reported in 2018 the case of a 36-year-old woman with alcoholic liver cirrhosis. She succumbed to endocarditis caused by *Lactobacillus rhamnosus* GG (LGG), even after receiving treatment with penicillin and gentamicin [121]. Kothari et al. suggested that sepsis may be related to bacterial translocation across a compromised intestinal barrier. In healthy immunocompetent individuals, translocated bacteria are typically trapped and destroyed within mesenteric lymph nodes. However, this defensive mechanism is impaired in conditions such as heart failure, where gut barrier function is compromised [122]. In summary, while the advantages of probiotics are undeniable and extensively supported by evidence, the potential for adverse effects, though rare, does exist. Therefore, clinicians should carefully weigh the risks and benefits when selecting probiotic treatments and closely monitor patients following administration.

**Fig. 3:**
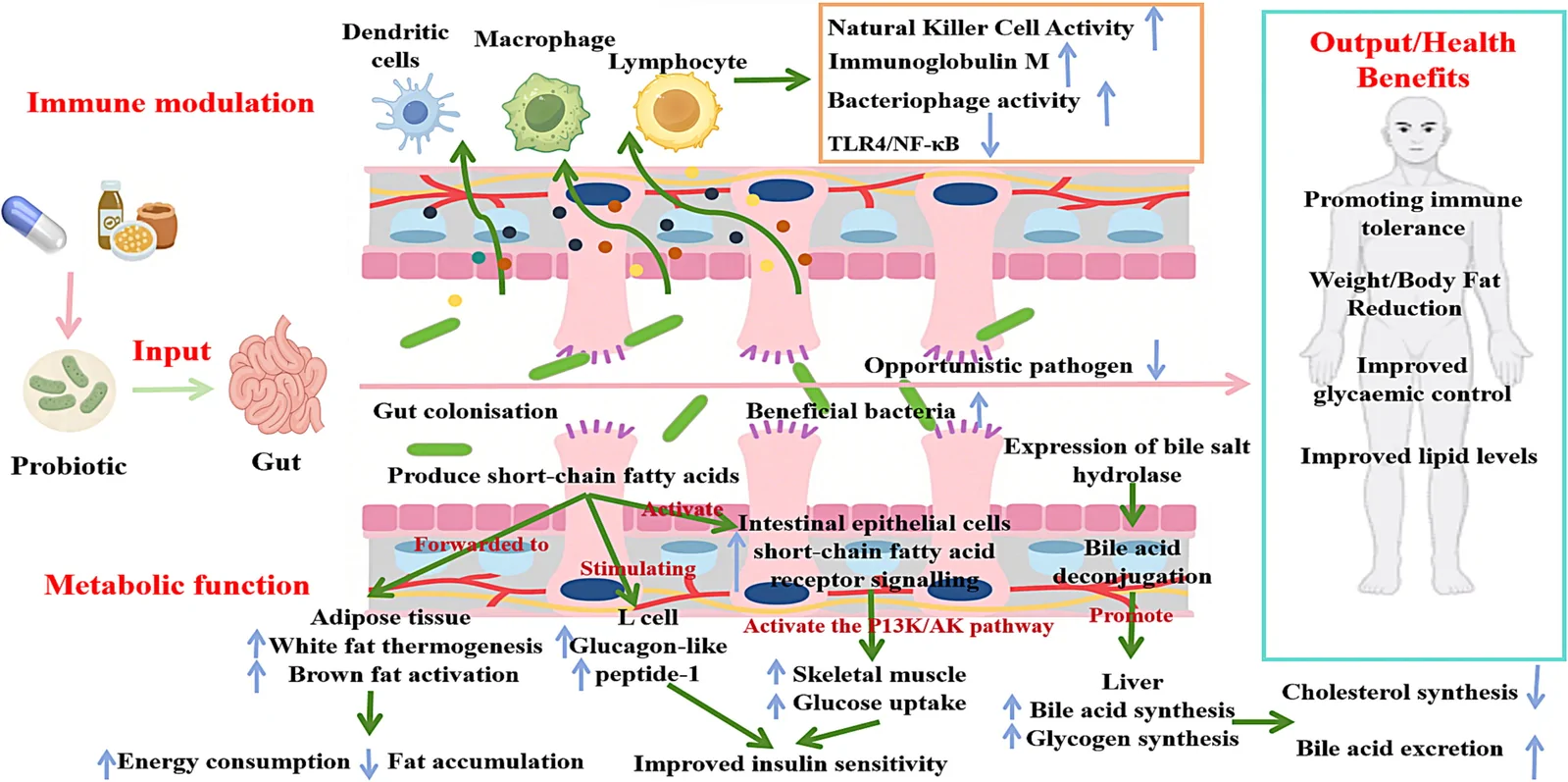
Probiotics: From Microorganisms to Health Benefits. Light blue arrows pointing upward indicate a promoting effect, while those pointing downward indicate an inhibitory effect.

In summary, probiotics extend beyond alleviating GI symptoms to provide benefits in hypercholesterolemia, lipid homeostasis, and T2D. Through fine-tuning of host immunity, these microorganisms aid in the prevention and management of immune-mediated and inflammatory conditions, including IBD, IBS, infectious diarrhea, infant colic, and certain cancers (Fig. 3). Nevertheless, the molecular mechanisms underlying probiotic-immune interactions remain incompletely characterized. Further preclinical and clinical studies are essential to elucidate these pathways and to identify novel probiotic strains that may exceed current immunomodulatory and metabolic benefits.

### Prebiotic

Probiotics are defined as viable microorganisms that, when administered in adequate amounts, offer measurable health advantages to the host. Prebiotics function as selective substrates that facilitate the stabilization and growth of beneficial microbial communities already present within the gut, thus aiding in the preservation of their ecological niche. The co-administration of probiotics and prebiotics—referred to as synbiotics—can enhance and prolong these beneficial effects [123]. Recent advances in encapsulation technology demonstrate this synergistic potential. In one study, various prebiotics were incorporated as wall materials in water-in-oil-in-water (W/O/W) emulsions. Subsequent spray-drying and reculture assays revealed that the survival rate of LGG increased from 43.23 % to 65.16 %. Fluorescence and scanning electron microscopy confirmed the structural integrity of the double-emulsion microcapsules and the well-preserved subcellular architecture of the bacteria [124]. Beyond improving probiotic viability, prebiotics exert direct ecological and epithelial influences. Upon reaching the colon, they are selectively fermented by beneficial taxa, thereby reinforcing chemical, mechanical, biological, and immunological barrier functions. Simultaneously, they stimulate enterocytes to enhance mucosal renewal, increase mucus secretion, and strengthen intercellular junctions, collectively supporting the integrity of both the epithelial and mucosal layers [125].

Per the International Scientific Association for Probiotics and Prebiotics (ISAPP), a dietary prebiotic is defined as a selectively fermented substrate that brings about specific, beneficial alterations in the composition and/or activity of the GI microbiota, thereby imparting health benefits [126]. To be classified as a prebiotic, a compound must fulfill three criteria: (1) resist gastric acidity and breakdown by mammalian enzymes, (2) remain unabsorbed within the upper GI tract, and (3) be selectively used by host microorganisms associated with health benefits. Chemically, prebiotics fall into four broad categories: fermentable carbohydrates, phenolic compounds, plant-derived phytochemicals (e.g., curcumin, naringenin, hesperetin), and polyunsaturated fatty acids (PUFAs) such as linoleic acid, docosahexaenoic acid, and eicosapentaenoic acid. Among these, carbohydrate-based prebiotics—including short-chain oligosaccharides (e.g., inulin-type fructans, galacto-oligosaccharides (GOS), arabinoxylans, *xylo*-oligosaccharides (XOS), and human milk oligosaccharides) and longer polysaccharides (e.g., β-glucans, isomaltodextrin, and resistant starch)—remain the most extensively studied and widely applied, owing to their consistent efficacy in promoting beneficial gut bacteria [127].

Prebiotics function by serving as selective substrates for commensal microorganisms, thereby reshaping the gut ecosystem and influencing host physiology. Dietary introduction of these indigestible compounds represents a straightforward strategy for establishing or restoring a healthy microbiota, particularly during states of dysbiosis. Accumulating evidence supports a wide range of health benefits associated with prebiotic intake, including maintenance of intestinal barrier integrity, favorable modulation of blood lipid profiles, and anti-diabetic, anti-inflammatory, and antihypertensive effects [128]. Among emerging prebiotic candidates, anthocyanins—flavonoid pigments abundant in deeply colored fruits, vegetables, and whole grains—have garnered significant interest. Systematic reviews of trials in T2DM indicate that anthocyanin supplementation for ≥ 4 weeks significantly reduces glycated hemoglobin (HbA1c), fasting plasma glucose, 2-hour postprandial glucose, triglycerides, and low-density lipoprotein (LDL) cholesterol [129]. Beyond improving glycemic control, anthocyanins enhance endothelial function, reduce uric acid levels, and favorably alter microbial metabolites in both prediabetic and diabetic individuals, thereby mitigating T2DM complications [130]. Importantly, these cardiometabolic benefits are enhanced when anthocyanins are co-administered with prebiotics that improve their bioavailability and microbial metabolism. Clinical meal studies demonstrate this synergy: replacing white rice with anthocyanin-rich riceberry rice—which naturally contains fermentable fibers—slowed gastric emptying and attenuated postprandial glucose excursions in healthy volunteers [131]. Similarly, a low-dose synbiotic blend comprising inulin, anthocyanin, and rice-bran components improved glycemic and lipid parameters in T2DM patients [128]. Together, these findings underscore the therapeutic potential of combining antioxidant-rich plant pigments with established prebiotic fibers to optimize metabolic health.

Although prebiotics are indigestible soluble fibers that remain intact throughout the small intestine, they play a critical role in modulating host cell physiology. Diets rich in these fibers have been increasingly associated with enhanced immune competence [125]. Asthma, a common airway disorder driven in part by immune dysregulation, may be ameliorated by interventions that recalibrate immune responses, such as probiotics and prebiotics. In a murine model of ovalbumin-induced asthma, BALB/c mice receiving either probiotics (*Lactobacillus acidophilus* LA-5*,* LGG*, Bifidobacterium animalis* ssp. *Lactis* BB-12*)* or prebiotics (fructo-oligosaccharides (FOS) and GOS) exhibited reduced airway hyperresponsiveness, decreased eosinophil infiltration in bronchoalveolar lavage fluid, and lower serum IgE/IgG1 levels. Both treatments also suppressed IL-17, glutamic-pyruvic transaminase (GPT), and eosinophil peroxidase activity, while reducing goblet cell hyperplasia, mucus overproduction, and peribronchial/vascular inflammation. Probiotic administration downregulated toll-like receptor 4 (TLR4) and C–C motif chemokine ligand 11 (CCL11) expression, whereas both probiotics and prebiotics upregulated the anti-inflammatory cytokine IL-38. Furthermore, both interventions inhibited T helper type 2 (Th2) cytokines (IL-4, IL-5, IL-13), alarmins (IL-25 and IL-33), leukotrienes, and key signaling molecules (Protein kinase B (AKT), NOD-like receptor protein 3 (NLRP3), Nuclear factor-κB (NF-κB), Myeloid differentiation factor 88 (MyD88), Mucin 5AC (Muc5AC)). Prebiotics alone also reduced peribronchial inflammation and phosphatidylinositol 3-kinase (PI3K) gene expression. Neither treatment altered glutamic oxaloacetic transaminase (GOT), thymic stromal lymphopoietin (TSLP), IL-8, eotaxin, or C–C motif chemokine ligand 24 (CCL24) levels. These results collectively indicate that targeted probiotic and prebiotic interventions can promote immune tolerance and mitigate allergic-inflammatory pathways in asthma [132].

Prebiotics selectively nourish beneficial gut microbes, stimulating the production of various bioactive molecules—primarily SCFAs such as butyrate, propionate, and acetate, along with antimicrobial peptides [133]. These metabolites collectively acidify the colonic environment, creating conditions that favor probiotic persistence, proliferation, and mucosal adhesion. Maintenance of an optimal luminal pH is essential for supporting the adhesive capacity of probiotic strains. SCFAs produced through prebiotic fermentation activate G-protein coupled receptors (GPCRs) on intestinal epithelial and immune cells. Ligand-receptor binding initiates downstream signaling cascades, including the mitogen-activated protein kinase (MAPK) pathway, leading to upregulation of epithelial antimicrobial peptide expression. Concurrently, SCFAs modulate the activity of T-cells, dendritic cells, and macrophages, promoting an anti-inflammatory cytokine profile. Through these integrated mechanisms, SCFAs reinforce epithelial barrier function, enhance mucosal immunity, and contribute to overall gut homeostasis [134].

**Fig. 4:**
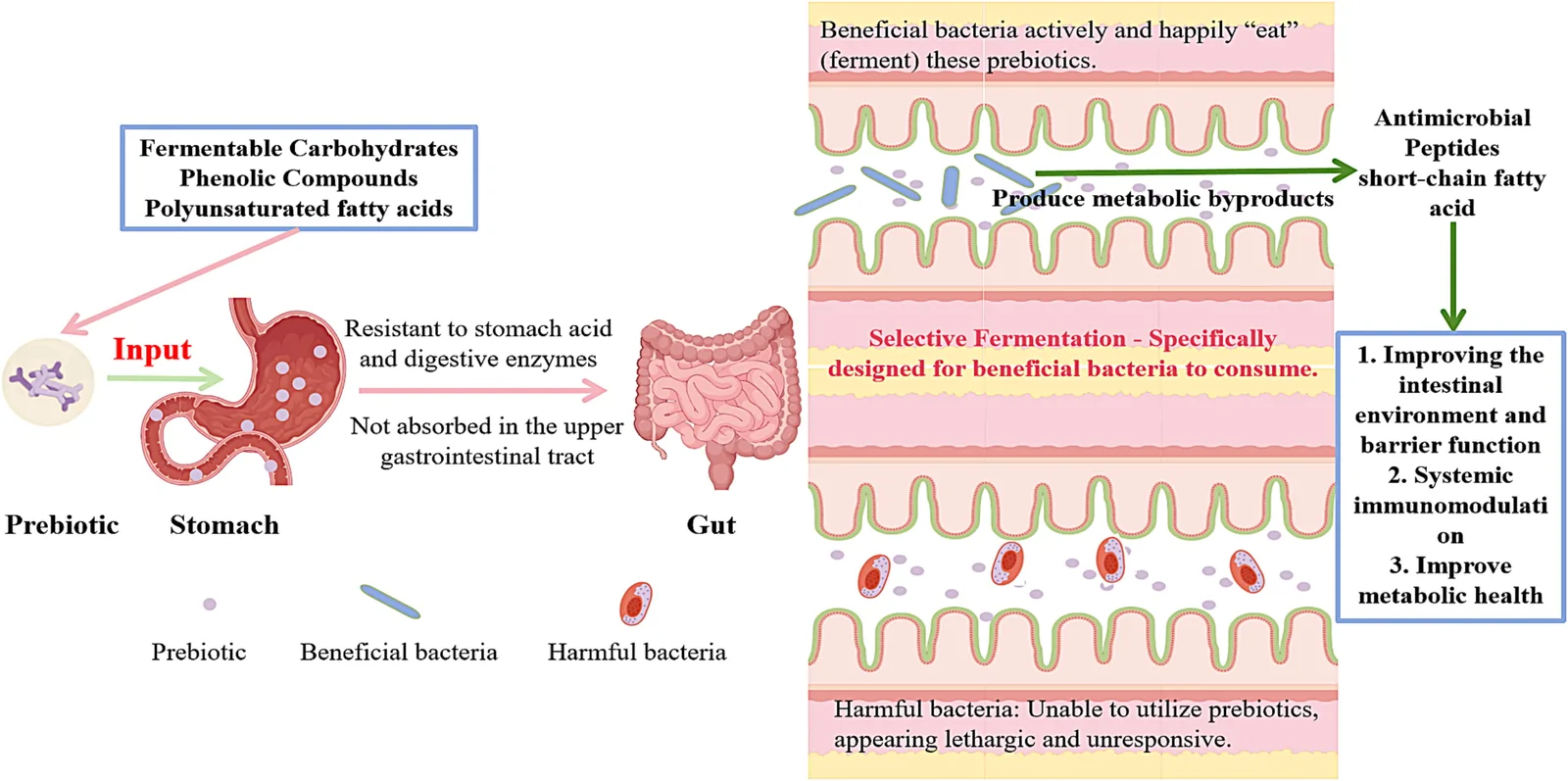
Prebiotics: Sources, Mechanisms and Health Benefits. Light blue arrows pointing upward indicate a promoting effect, while those pointing downward indicate an inhibitory effect.

Classical prebiotics include inulin, FOS, GOS, and lactulose, while a second generation of candidates—such as XOS, raffinose oligosaccharides, isomaltose, polyols (lactitol, xylitol, mannitol), structural polysaccharides (pectin, β-glucan, lignin), and resistant starches—await further validation. Current research has expanded the prebiotic concept to include phenolics, carotenoids, PUFAs, and certain vitamins [135]. Although their mechanisms of action are still being elucidated (Fig. 4), these novel agents demonstrate the capacity to support a stable and diverse microbiota. A deeper understanding of how each class functions will facilitate the development of targeted synbiotic strategies aimed at preventing or treating dysbiosis-associated disorders.

### Synbiotic

Continuous crosstalk between the human microbiome and the immune system is essential for maintaining physiological homeostasis [136]. Consequently, targeted modulation of microbial communities offers a promising approach to fine-tune immune responses and reduce infection susceptibility, generating growing interest in microbiota-based strategies for enhancing host defense mechanisms (Table 3) [137].

**Table 3:** Synbiotic pairing (probiotics and prebiotics) and experimental results.

Synbiotic	Probiotic	Prebiotic	Control group	Experimental results	References
Human randomized controlled trial	*Bifidobacterium lactis* HN019 and *Lactobacillus rhamnosus* HN001	Fructooligosaccharide	A sachet of placebo powder containing maltodextri	Compared to the placebo group, participants receiving synbiotic supplementation exhibited greater reductions in plasma C-reactive protein and interferon-gamma, along with larger increases in plasma IL-10 and stool secretory IgA.	[137]
	*Lactobacillus rhamnosus, Lactobacillus reuteri, and Bifidobacterium infantis*	3 % fructooligosaccharides, and other ingredients including sunflower oil, medium-chain triglyceride, silicon dioxide, and natural flavoring.	Placebo was prepared in packages with the same shape and color as the synbiotic.	The incidence of NEC decreased significantly. Additionally, feeding intolerance was less frequent in this group. Furthermore, consumption of the synbiotic was associated with significant weight gain in infants.	[143]
Animal experimentation	*Lactobacillus suilingensis* AF91-01CMCA	Inulin	AD mice (Pure water treatment), inulin treatment group, and AF91 treatment group	Indole lactic acid (ILA) synthesis was downregulated in AD patients. Synbiotic treatment outperformed probiotic treatment alone in enhancing tryptophan metabolism, and increasing ILA biosynthesis. Increased ILA could reduce Aβ. accumulation and significantly alleviate cognitive impairment in female AD mice by inhibiting neuroinflammation through activation of the aryl hydrocarbon receptor (AhR) signaling pathway.	[152]
	*Lactobacillus rhamnosus* S 82	Xylo-oligosaccharide	Normal group, the model group, the probiotic group, the prebiotic group, and the drug simvastatin group	The therapeutic effect of synbiotics on hypercholesteremic mice was investigated, and the results demonstrated that synbiotics notably decreased the levels of total cholesterol (TC), triglycerides (TG), and low-density lipoprotein cholesterol (LDL-C) in the physical and chemical indexes, and mitigated liver damage.	[153]
*In vitro* experimentation	*Limosilactobacillus fermentum* K73	Gh-oleic palm oil, and sweet whey	/	The results revealed a decrease in the abundance of genera like *Bacteroides* and *Dorea* in ASD-associated samples after the treatment with the synbiotics. Furthermore, the analysis of short-chain fatty acids and serotonin indicated that the synbiotic intervention resulted in an elevated butyric acid and microbial serotonin synthesis, alongside a decrease in propionic acid.	[156]

A synbiotic refers to a mixture consisting of viable microorganisms and substrate(s) that can be selectively utilized by the host’s microorganisms, thereby imparting health benefits to the host. There are two acknowledged types of synbiotics. A complementary synbiotic is composed of a probiotic and a prebiotic; the two work in conjunction to provide one or more health benefits but do not rely on interdependent functions, and each component must be used at doses proven effective when administered individually. A synergistic synbiotic, by contrast, includes a substrate that is selectively utilized by the co-administered viable microorganism(s) [138]. Despite demonstrated improvements in inflammatory markers in disease models and clear theoretical advantages, evidence for the actual efficacy of synbiotics—particularly their immunomodulatory effects in healthy populations—remains limited and inconclusive. This scientific uncertainty stands in stark contrast to the growing popularity of synbiotic products as “immune support” strategies among the general population, particularly self-reported healthy individuals. Therefore, to clarify their true efficacy, more systematic and rigorous clinical studies involving healthy subjects are urgently needed [139].

Strains from *Lactobacillus* and *Bifidobacterium* are among the most widely studied probiotics with immunomodulatory properties. Notably, *Bifidobacterium lactis* HN019 and *Lactobacillus rhamnosus* HN001—two well-characterized immunomodulatory strains—exhibit enhanced efficacy when combined with fructooligosaccharides [140]. Leveraging this synergistic potential, a double-blind, placebo-controlled trial was conducted. Healthy volunteers were given an 8-week regimen of a synbiotic that combines these two probiotics with a prebiotic. When compared to the placebo group, the synbiotic group exhibited more notable decreases in plasma C-reactive protein and interferon-gamma, along with larger increases in plasma IL-10 and stool secretory IgA. Additionally, synbiotic intake led to a higher abundance of beneficial bacteria—such as *Clostridium sensu stricto* 1, *Lactobacillus*, *Bifidobacterium*, and *Collinsella*—and improved several functional pathways related to amino acid and SCFA biosynthesis. It also reduced levels of the potentially pro-inflammatory genus Parabacteroides when contrasted to baseline. Notably, changes in anti-inflammatory markers (IL-10 and IgA) were significantly correlated with the microbial changes brought about by synbiotic supplementation [137].

Necrotizing enterocolitis (NEC) is a multifactorial condition predominantly seen in very low birth weight (VLBW) infants [141], with pathogenesis linked to disrupted bacterial colonization [142]. A double-blind, randomized controlled trial assessed the impacts of PediLact®—an oral multi-strain synbiotic formulated with *Lactobacillus rhamnosus*, *Lactobacillus reuteri*, and *Bifidobacterium infantis*—on nutritional outcomes and the occurrence of NEC in VLBW neonates. The study recruited 118 infants from neonatal intensive care units, who were randomly allocated in a 1:1 ratio to receive either the synbiotic or a placebo until they attained full enteral feeding. Results indicated that the synbiotic group had a notably lower rate of NEC and diminished feeding intolerance. Furthermore, these infants showed a higher weight gain (roughly 40 g), a shorter period required to reach full enteral feeding, and a hospitalization duration reduced by around 3 days. No severe adverse events were documented in the study [143].

While these findings indicate that synbiotics may serve as a secure and efficacious approach for supporting immune function in healthy adults and reducing the occurrence of NEC in extremely low birth weight infants, it is important to note that the relatively small sample size and single-center study setup limit the generalizability of these outcomes to wider adult populations. Larger cohort studies with more diverse participant groups are required to validate these preliminary observations.

Alzheimer's disease (AD) is a neurodegenerative disorder characterized by memory loss and cognitive decline [144]. Pathological hallmarks include amyloid-β (Aβ) deposition, Tau protein pathology, and neuroinflammation, although the precise etiology remains incompletely understood [145]. Emerging evidence indicates that alterations in the gut microbiota are closely linked to AD pathogenesis, as evidenced by reduced microbial diversity and compositional shifts in affected individuals [146]. These changes are associated with distinct profiles of microbial metabolites, particularly those derived from tryptophan metabolism [147]. Among these, microbial tryptophan catabolites (MTCs) have attracted significant attention due to their roles in regulating host metabolism, immunity, and neurodevelopment [148]. Studies report decreased fecal levels of indole derivatives such as indole carboxylic acid and 3-hydroxyethyl indole in AD patients, alongside elevated indole-3-pyruvate acid (IPyA) [149]. Notably, plasma levels of indole lactic acid (ILA) are significantly reduced in AD patients compared to cognitively normal controls [150]. Similar disruptions, including reduced levels of gut-derived indole-3-propionic acid (IPA), have been observed in animal models [151]. Current evidence suggests that probiotic supplementation can modulate gut microbiota structure and tryptophan metabolism, increasing indole compound levels and potentially aiding in AD prevention and treatment. Consistent with this, synbiotic supplementation has been shown to regulate microbial tryptophan metabolism in AD mouse models, increasing the abundance of ILA biosynthetic genes and elevating ILA concentrations *in vivo*. However, such interventions may also affect other indole metabolites, warranting further investigation into their specific roles in AD pathology. Additionally, synbiotic supplementation reduces Aβ accumulation, suppresses neuroinflammation, and ameliorates cognitive deficits in female AD mice [152]. It should be noted, however, that these findings are derived from a single-sex animal model, leaving the efficacy in males and the potential influence of sex on the intervention outcome unexplored. Future studies incorporating both sexes and different age groups are crucial to determine whether the benefits of synbiotics are generalizable or subject to population heterogeneity, which will be essential for guiding targeted applications in humans.

Changes in dietary patterns and lifestyle have contributed to rising cholesterol levels in many populations. Excessive intake of high-cholesterol foods disrupts lipid metabolism, and substantial evidence supports the cholesterol-lowering effects of probiotics and prebiotics. Synbiotic combinations often demonstrate greater efficacy than either component alone. A novel screening strategy focused on prebiotics identified a synbiotic combination of *Lactobacillus rhamnosus* S 82 and XOS, selected through iterative enrichment from young donors’ microbiota. *In vitro* simulations using glucose and XOS as carbon sources indicated that this synbiotic may lower cholesterol by downregulating expression of the Niemann-Pick C1-like 1 (NPC1L1) and acetyl-coenzyme A (CoA) acetyltransferase 2 (ACAT2) genes, upregulating ATP binding cassette subfamily G member 8 (ABCG8), and modulating intestinal microbiota composition through adsorption and metabolic activities [153]. These findings support the potential of synbiotics in preventing hypercholesterolemia and provide a theoretical basis for developing new synbiotic products.

Autism spectrum disorder (ASD), a neurodevelopmental condition, has been associated with disruptions in gut microbiota composition, showing dysbiosis relative to neurotypical children [154]. Some studies have focused on modulating the gut microbiota in children with ASD using specific probiotics and compounds, aiming to correlate these changes with behavioral improvements [155]. One study assessed the impacts of spray-dried synbiotics—formulated with *Limosilactobacillus fermentum*, high-oleic palm oil, and sweet whey—on the gut microbiota of Colombian children with and without ASD. Utilizing *in vitro* digestion and fermentation models, the production of SCFAs, including lactic, acetic, propionic, and butyric acids, as well as serotonin, was quantified. The structure of the microbial community was evaluated through 16S rRNA gene sequencing. Treatment with the synbiotic led to a decreased abundance of genera such as *Ruminococcus*, *Dorea*, and *Bacteroides* in ASD samples, while increasing the levels of *Lactobacillus*, *Streptococcus*, and *Anaerostipes*. Additionally, it resulted in elevated levels of butyric acid and microbial serotonin, along with reduced propionic acid—changes that are regarded as beneficial in the context of ASD [156].

**Fig. 5:**
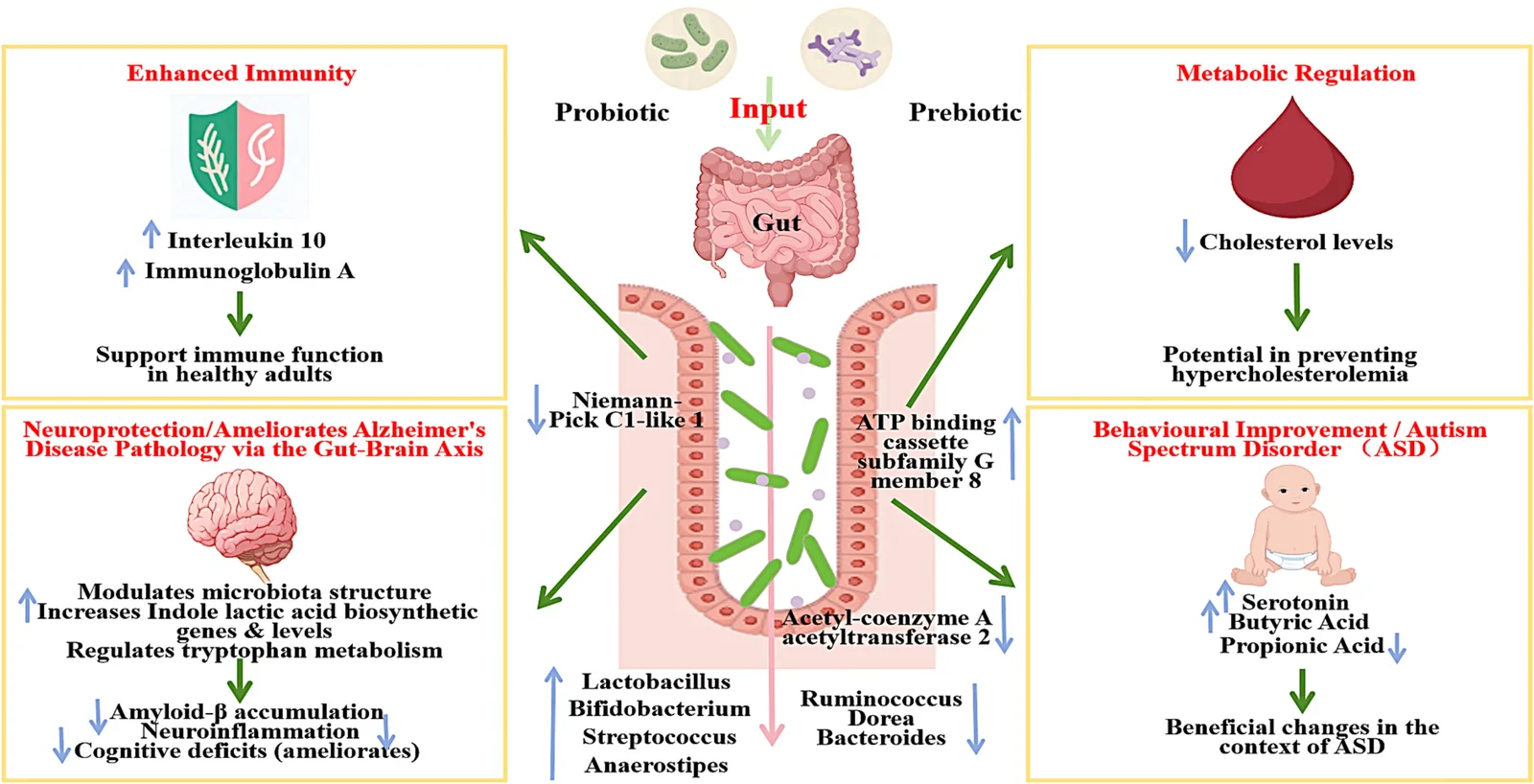
Synbiotics: Roles in Immunity, Neuroprotection, Metabolism, and Behavioral Health. Light blue arrows pointing upward indicate a promoting effect, while those pointing downward indicate an inhibitory effect.

Synbiotics confer health benefits by immune stimulation and pathogen suppression (Fig. 5). Growing interest in the applications of probiotics, prebiotics, and synbiotics in healthcare and consumer products is tempered by the limited breadth of research, which remains concentrated on a small number of strains and substrates. Although synbiotics represent a promising integration of probiotics and prebiotics, current research is often fragmented and superficial. Many studies adopt a “random pairing” approach without verifying strain-specific substrate utilization. The clinical ineffectiveness of synbiotics may partly stem from such “random pairing.” One clinical trial observed that synbiotic intervention provided only limited improvement in diabetes-related biomarkers, without enhancing glucose metabolism [157]. In another trial involving 120 prediabetic adults, researchers compared probiotics ( *Bifidobacterium bifidum*, *B. lactis*, *B. longum*, and *Lactobacillus acidophilus*, 1.5 × 10^9^ CFU each) with synbiotics (the same probiotics plus inulin). After six months, the synbiotic intake did not successfully regulate gut microbial composition [158]. To overcome this bottleneck, future research must shift from “random pairing” to “rational design”. We propose establishing a standardized stepwise validation pathway: first, using *in vitro* fermentation models to screen candidate combinations and identify pairs exhibiting specific synergistic effects; then, validate their efficacy and mechanisms using advanced models like intestinal organ-on-a-chip under simulated physiological conditions; finally, confirm their health benefits through carefully designed small-scale human trials. Through this stepwise strategy can truly effective synbiotics can be efficiently identified, advancing them from concept to reliable clinical application.

### Postbiotic

In the twenty-first century, growing consumer interest in holistic nutrition and functional foods as means to address suboptimal health [159] has brought probiotics and postbiotics into scientific focus. Advances in microbial science have expanded the applications of probiotics beyond dietary supplements to include infant formula [160], agriculture, aquaculture, and clinical medicine [161]. Defined as viable, non-pathogenic microorganisms that confer measurable health benefits, probiotics are known to modulate immune function, secrete antimicrobial compounds, competitively exclude pathogens, and regulate gut motility and electrolyte absorption [162]. However, limitations such as variable product quality, limited shelf stability, inconsistent efficacy, and safety concerns in immunocompromised populations restrict their broader application—particularly under harsh processing or storage conditions such as pasteurization or baking [163].

To tackle these challenges, postbiotics—defined as “a preparation of inanimate microorganisms and/or their components that confers a health benefit on the host” [164]—have emerged as a highly promising alternative. The definition of postbiotics encompasses inactivated bacteria and cellular components (such as pili, cell wall components, or other structural elements), as well as soluble bioactive factors (metabolic byproducts or metabolites) generated by microorganisms during their growth and fermentation processes [164], [165], [166]. These shelf-stable formulations include microbial cell-wall fragments (e.g., teichoic acids, peptidoglycan, and exopolysaccharides) and metabolites (e.g., bacteriocins), which contribute to host health by (i) inhibiting pathogens, (ii) reinforcing epithelial barrier integrity, and (iii) modulating immune and inflammatory responses. Already incorporated into fermented foods, postbiotics are increasingly recognized as therapeutic options for suboptimal health states [167], particularly GI symptoms such as bloating and diarrhea. As such, they serve both as complements to probiotics and as catalysts for the development of integrated health strategies (Table 4) [168].

**Table 4:** Some postbiotics: components, definition, stability, and relevant evidence.

Postbiotic	Components	Definition	Stability	Relevant evidence	References
Bacterial Components	Lipopolysaccharides (LPS)	Made of an O-specific antigen, a core oligosaccharide, and a multi-acylated lipid A	High (heat, acid, and alkali stability)	Penta-acylated LPS derived from *R. Sphaeroides* can directly oppose deleterious changes in gut barrier function, adipose tissue inflammation, insulinogenic potential and blood glucose control caused by *E. Coli* LPS	[198], [238]
	Muropeptides	The building blocks of peptidoglycan, and they are recycled by both Gram-positive and Gram-negative bacteria during cell division	Medium-high (depending on specific structure)	Muramyl dipeptide (MDP) is the minimal bioactive muropeptide motif and a ligand for NOD2. MDP intraperitoneal injection improves blood glucose control and lowers insulin resistance in various models of obesity and metabolic endotoxemia	[239], [240]
	Flagellin	Flagellin is part of the bacterial locomotor appendage flagellum used in motility	Medium-high (Recombinant Production Capable)	To boost immunoglobulins targeting flagellin, repeated flagellin intraperitoneal injections in mice elicited a mucosal flagellin-specific IgA antibody response that can alter the intestinal bacterial profile, reducing flagellin expression to prevent microbial encroachment, lower inflammation, and protect against diet-induced obesity	[241], [242]
	Lipoteichoic Acids(LTA)	Amphiphilic macromolecules that contain hydrophobic and hydrophilic components in the cell wall of Gram-positive bacteria	High	Oral LTA supplementation with heat-killed *Lactobacillus paracase*i strain D3-5 lowered glucose intolerance, insulin resistance, and hepatic steatosis and adipocyte size in diet-induced obese mice	[243], [244]
Metabolites	Short-Chain Fatty Acids (SCFAs)	Fermentation of nondigestible carbohydrates by the microbiota can result in production of SCFAs, such as acetate, propionate, and butyrate	Medium	Acetate, propionate, and butyrate induce a metabolic shift from lipogenesis to fatty acid oxidation in adipose and liver tissue by downregulating peroxisome proliferator-activated receptor gamma and subsequent activation of uncoupling protein 2-AMP-activated protein kinase (AMPK)-acetyl-CoA carboxylase signaling pathway	[245], [246]
	Trimethylamine Oxide (TMAO)	Certain species of gut commensals metabolize nutrients such as choline and phosphatidylcholine in our foods to produce trimethylamine, which is absorbed and then converted into TMAO by flavin-containing monooxygenase 3 in the liver	High (Water-soluble, chemically stable)	TMAO intake lowers transcripts involved in insulin signal transduction and upregulates genes involved in glucose production via gluconeogenesis in the liver	[247], [248]
	D-Lactate	The chemical enantiomer of L-lactate that is involved in energy and carbon transfer through the Cori cycle	High (Chemically stable)	D-lactate can influence immune function in the intestine and liver, which is positioned to alter endocrine control of metabolism. Morita et al showed that gut microbial-derived D- and L-lactate promote antigen uptake by CX3CR1^+^ myeloid cells in the gut through activation of G protein-coupled receptor 31 (GPR31)	[249], [250]
	Succinate	One of the intermediates of the tricarboxylic acid cycle (TCA) and the link between TCA and the mitochondrial respiratory chain in the host	High	Increasing succinate in the gut does not increase succinate in the blood, showing that succinate is compartmentalized to the intestine for use in local gluconeogenesis. Succinate-induced intestinal gluconeogenesis increases glucose in the portal vein, which actually improves whole blood glucose homeostasis and decreases hepatic glucose production	[251], [252]

Chronic constipation is a common GI disorder linked to intestinal inflammation that severely compromises patients' quality of life [169]. While probiotics are limited by host-related factors and stability issues [170], postbiotics stand out as a safe, stable alternative—boasting advantages such as well-characterized structures, no risk of antibiotic resistance, and ease of storage [171]. Multiple lines of evidence have verified the effectiveness of postbiotics. For instance, metabolic products derived from *Lacticaseibacillus paracasei* can strengthen the intestinal barrier function and modulate water-sodium metabolism in mice with constipation [172]; meanwhile, heat-inactivated *Bifidobacterium bifidum* MIMBb75 has been shown to alleviate abdominal pain in patients diagnosed with IBS [173]. A randomized, double-blind, placebo-controlled crossover trial was carried out to evaluate Probio-Eco—a postbiotic product fermented by *Lacticaseibacillus paracasei* Zhang, *Lactiplantibacillus plantarum* P-8, and *Bifidobacterium lactis* V9, with soy protein, skimmed milk powder, and sodium citrate serving as fermentation substrates. A total of 110 adults suffering from chronic constipation were enrolled in this study. After a 3-week intervention with Probio-Eco, the results demonstrated significant improvements in constipation symptoms, stool straining severity, and worry-related scores among the participants. Analyses of gut microbiota and metabolomics indicated distinct changes in specific microbial communities, as well as variations in the levels of succinate, tryptophan derivatives, deoxycholate, propionate, butyrate, and cortisol—all of which showed a correlation with the alleviation of constipation symptoms. Additionally, experiments using a loperamide-induced constipation mouse model further confirmed that Probio-Eco and its bioactive components (including succinate, 3-indoleacrylic acid, and 5-hydroxytryptophan) exert a constipation-relieving effect. The underlying mechanisms involve stimulating the secretion of mucin-2, regulating intestinal transport hormones, and boosting anti-inflammatory responses. These comprehensive findings not only highlight the effectiveness of Probio-Eco but also provide solid evidence for its clinical application in the management of chronic constipation [174].

*Aspergillus oryzae*, a fungus long integral to East Asian fermentation processes [175], is now being investigated for its postbiotic potential. Recent studies indicate that cell-free extracts of *A. oryzae* confer health benefits in animal models, suggesting broad applicability in promoting well-being [176]. In *Drosophila*, such an extract enhanced tolerance to heat stress [177]. Dairy cows subjected to heat stress showed increased milk yield and reduced inflammation when fed the same preparation, while calves demonstrated improved intestinal barrier function and energy efficiency, albeit without systemic anti-inflammatory changes [178]. In sows, supplementation during gestation and lactation reduced maternal weight loss, though no significant effects on litter performance were observed [179]. Together, these findings indicate that *A. oryzae*-derived postbiotics can enhance thermotolerance and physiological resilience across species.

Postbiotics of microbial origin are also garnering interest in oncology due to their ability to benefit the host, reduce inflammation, and directly inhibit tumor cells. Among these, butyrate—a quantitatively dominant microbial metabolite—has been the most extensively studied for its anti-cancer properties [180]. Pancreatic cancer (PC), characterized by a five-year survival rate of less than 10 % [181], often presents with metastatic spread at diagnosis, and current serum biomarkers lack sensitivity [182]. Butyrate-producing gut commensals such as *Ruminococcus* and *Faecalibacterium* contribute to signaling modulation, autophagy, and cell-cycle regulation; reduced levels of fecal or circulating butyrate have been correlated with PC development [183]. Consequently, butyrate is being explored as a potential early biomarker prior to metastasis [184]. Beyond diagnostics, it exerts anti-tumor effects through mechanisms including histone deacetylase inhibition, induction of mitochondrial apoptosis, activation of extrinsic death-receptor signaling, promotion of cellular differentiation, and broad anti-inflammatory activity [185].

The discovery and characterization of new postbiotics not only shed light on the mechanisms underlying the actions of probiotics but also drive the development of innovative therapeutic approaches for various diseases. In a study carried out by Ruopeng Yin and colleagues, a postbiotic derived from the gut commensal bacterium *Lactobacillus johnsonii* was shown to exert a protective effect against ethanol-induced gut microbiota dysbiosis, mucosal impairment, and hepatic damage. Specifically, the administration of this postbiotic—prepared from heat-killed *L. johnsonii* (HKLJ)—upregulates the expression of intestinal lysozymes. This, in turn, boosts the production of immunoregulatory ligands from HKLJ. These elevated ligands further activate nucleotide-binding and oligomerization domain-containing 2 (NOD2)-related signaling pathways in dendritic cells, facilitating the sorting of cellular lysozymes and the subsequent production of immune cytokines. This HKLJ-triggered regulatory loop plays a pivotal role in mediating its therapeutic effects against alcoholic liver disease (ALD) [186]. It is worth highlighting that the immunoregulatory components derived from *L. johnsonii*, as well as their regulatory pathways beyond the NOD2 axis, deserve further exploration. Additionally, the above-mentioned findings are based on animal studies and thus need to be validated through clinical trials.

*Escherichia coli Nissle 1917* (EcN), a Food and Drug Administration (FDA)-approved probiotic for pediatric acute diarrhea, produces bacteriocins and SCFAs that inhibit pathogens and stimulate gut motility, while competitively excluding harmful microbes to support microbial diversity and intestinal health [187], [188], [189]. However, genomic analyses indicate potential risks for immunocompromised individuals, as EcN is capable of synthesizing colibactin—a genotoxin associated with DNA damage and colorectal carcinogenesis [190]. Since viability is not always necessary for beneficial effects [191], research attention has shifted toward postbiotic formulations—heat or chemically inactivated EcN or its components—which retain efficacy while eliminating risks related to infection or bacterial translocation [192]. Recent evaluations of postbiotic preparations derived from *Escherichia coli strains* CEC15 (CEC15) and EcN in a murine model of 5-Fluorouracil (5-FU)-induced intestinal mucositis demonstrated that postbiotic CEC15 was similarly effective to its live counterpart, with certain results even indicating superior efficacy of the postbiotic preparation. In contrast, the postbiotic form of EcN outperformed the live bacteria in most tests, suggesting an advantage of postbiotics in the treatment of this strain [193].

LPS, a key component of the outer membrane in Gram-negative bacteria, is composed of three structural parts: an O-antigen, a core oligosaccharide, and lipid A. As a potent inducer of inflammatory reactions and metabolic abnormalities, it promotes the release of proinflammatory cytokines, including tumor necrosis factor-α (TNF-α), IL-6, and IL-1β [194]. LPS elicits inflammatory reactions by activating the TLR4/CD14 signaling pathway, which recruits Toll/interleukin-1 receptor (TIR) and MyD88 adaptor molecules to launch signaling cascades that propel proinflammatory responses. These responses can disrupt metabolism through multiple mechanisms, including cytokine production and activation of immune pathways such as the NLRP3 inflammasome, leading to insulin resistance and dysglycemia [195]. Genetic research lends support to this connection: for example, deletion of the CD14 gene in obese (ob/ob) mice led to a decrease in inflammatory markers within adipose tissue [196], while compromised CD14 signaling conferred protection to mice against glucose intolerance and inflammation induced by LPS and a HFD [197]. Notably, the immunologic and metabolic effects of LPS depend critically on its acylation state. Hexa-acylated LPS (e.g., from *E. coli*) activates TLR4, whereas penta-acylated forms (e.g., from *Rhodobacter sphaeroides*) can act as TLR4 antagonists. Anhê et al. demonstrated that administering R. sphaeroides LPS counteracted *E. coli* LPS-induced dysglycemia. Furthermore, chronic subcutaneous infusion of this penta-acylated LPS improved glycemic control, lowered insulin levels, and reduced adipose inflammation in obese mice. It also directly opposed the detrimental effects of *E. coli* LPS on gut barrier function and metabolism [198]. Thus, under-acylated LPS functions as a postbiotic capable of triggering metabolically favorable endotoxemia.

**Fig. 6:**
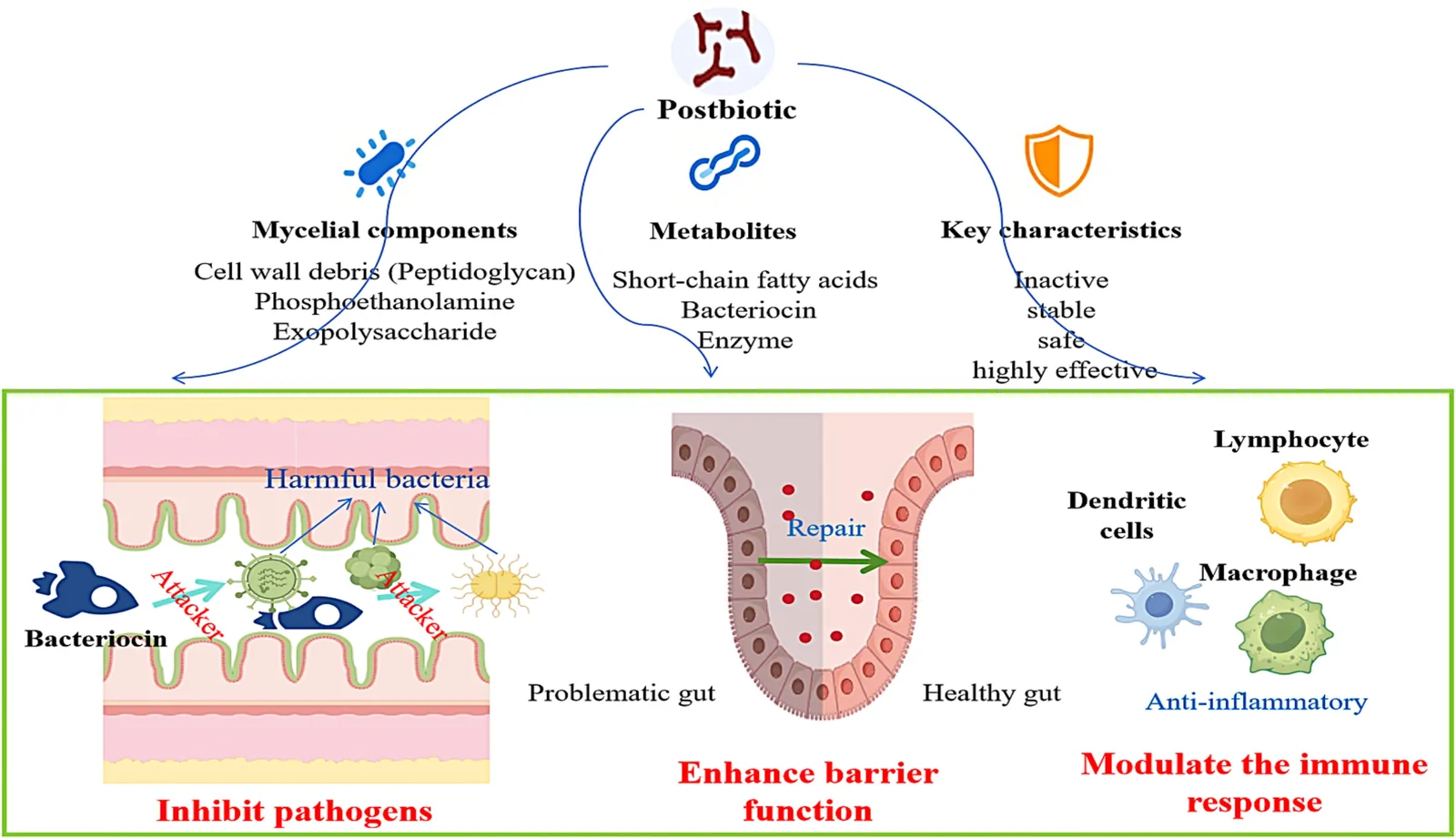
Postbiotics: The Next Generation of Health Solutions Beyond Probiotics. Problematic gut: gut microbiota imbalance and weakened intestinal barrier function. Healthy gut: balanced gut microbiota and intact intestinal barrier.

Postbiotics enhance epithelial barrier function, modulate immune activity, and exhibit antimicrobial properties, establishing them as valuable adjuncts or alternatives to probiotics—especially in managing GI disorders (Fig. 6). Nevertheless, as a nascent field, postbiotic research and development face challenges related to manufacturing and quality control. Standardized production protocols are essential to ensure batch-to-batch consistency, and robust methods are needed to reliably inactivate and quantify fungal-derived postbiotics with the same rigor applied to bacterial products. It is only through well-coordinated, interdisciplinary collaboration that dependable standards can be formulated—an essential step to unlock the full therapeutic potential of postbiotics in enhancing the health of both humans and animals.

### Summary

**Fig. 7:**
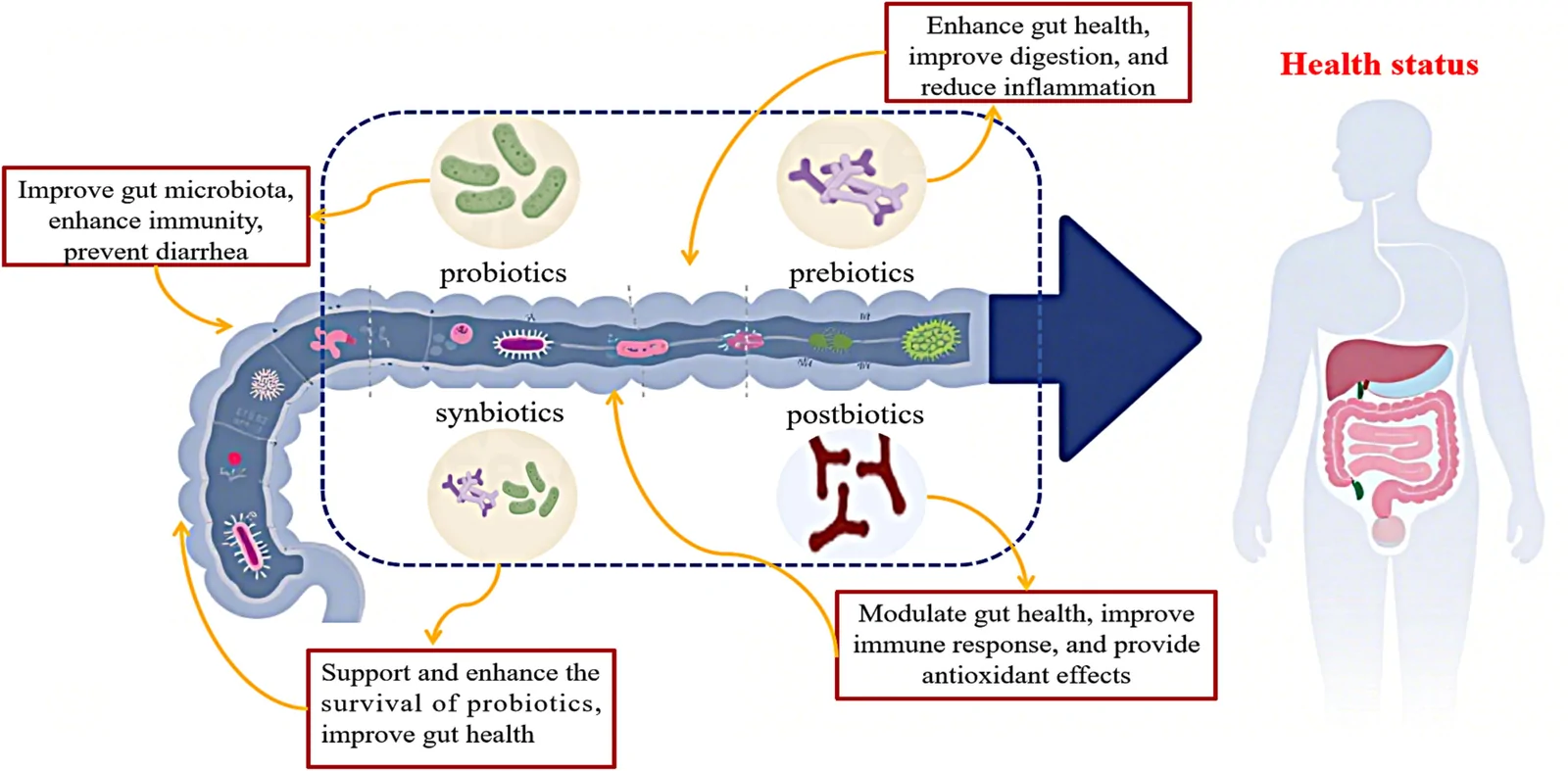
Summary of the role of Probiotics, prebiotics, synbiotics, and postbiotics.

In summary, prebiotics contribute to the restructuring and stabilization of the gut microbiota; probiotics alleviate GI symptoms and demonstrate efficacy in managing dyslipidaemia, hypercholesterolaemia, and T2D; synbiotics synergistically combine these benefits to enhance intestinal and immune function; postbiotics confer direct antimicrobial and anti-inflammatory effects; and despite associated risks, faecal microbiota transplantation (FMT) can reestablish microbial homeostasis (Fig. 7). While current evidence remains largely focused on digestive disorders, future investigations should prioritize expanding this focus to metabolic conditions such as cardiovascular disease and hyperuricaemia.

## Potential actors in gut microbiome therapeutics: bacteriophages and live dietary microorganisms

Numerous hypotheses have been proposed regarding the direct and indirect interactions of probiotics, prebiotics, synbiotics, and postbiotics with the microbiome. Despite an extensive body of research covering these interventions, the field continues to evolve with ongoing innovation. Beyond the well-established categories of probiotics, prebiotics, synbiotics, and postbiotics, several other substances, including bacteriophages, and active dietary microorganisms, are emerging as potential candidates for future inclusion within the probiotic-related spectrum [199].

### Bacteriophage

A bacteriophage is a virus that infects bacteria, replicating within host cells and rapidly generating numerous new infectious particles. During bacterial division, the quantity of bacteriophages (phages) can surge by 100 to 200 times. This robust ability for rapid replication, combined with their restricted host specificity, renders phages a highly promising therapeutic candidate for the treatment of bacterial infections [200].

The rise in antibiotic resistance, driven by excessive prescription practices, represents a critical challenge in modern healthcare. Bacteriophages provide a targeted alternative due to their specificity for particular bacterial species [201]. Their lytic enzymes—such as endolysins, exolysins, endopeptidases, endosialidases, and depolymerases—digest bacterial surface structures and extracellular polysaccharides, effectively disrupting biofilms [202]. This enzymatic activity can also expose receptors necessary for phage attachment [203]. Gram-positive pathogens are susceptible to degradation of peptidoglycan, wall teichoic acids, lipoteichoic acids, and flagella, whereas Gram-negative bacteria are vulnerable through LPS, pili, and capsules. Bacteria employ defense mechanisms, including physical barriers such as capsules or molecular systems like clustered regularly interspersed short palindromic repeats (CRISPR) associated proteins (Cas) system (CRISPR-Cas). Phage-derived proteins not only enhance pathogen-specific immune responses but can also resensitize bacteria to antibiotics [202].

The microbiome’s role in phage therapy is still an area of active research. In most cases, infectious agents can be considered invaders, either because they are not part of the normal microbiome at the infected site, have invaded sterile tissues, or have overgrown to pathogenic levels [204]. A commonly cited advantage of phage therapy is its ability to target specific pathogens without inflicting collateral damage on commensal microbiota. However, other members of the microbial community can influence phage efficacy; even phage-insensitive bacteria may adsorb phages on their surfaces or physically impede phage diffusion to new targets [205]. In essence, the microbiome is often viewed as a bystander rather than a central player in phage therapy.

In the context of *Clostridioides difficile* infection, numerous studies have shown that phages—especially those belonging to *C. difficile*—play an important role in boosting the efficacy of FMT [206]. Although the precise mechanisms remain unclear, phages are thought to contribute to disease resolution by modulating gut bacterial composition and functionality. Furthermore, fecal virome transplantation (FVT), which involves the administration of filtered donor stool containing gut viruses and associated metabolites, has been shown to be effective against *C. difficile* infection and induces long-term shifts in both bacterial and viral communities in patients [207]. Studies in mouse models have also indicated the therapeutic potential of FVT for metabolic disorders [208], dysbiosis [209], and NEC [210].

Given that the term “phage therapy” is already well established, there appears to be little need to introduce new terminology such as “phagebiotics” to describe the use of specific phages or phage cocktails for treating infections [211]. Nevertheless, if future applications involve directly targeting gut microbiota members—rather than pathogens—to confer health benefits in non-infectious contexts, the concept of “phage probiotics” may turn out to be useful.

In this regard, it is necessary to highlight several noteworthy issues. Although most studies confirm the high safety profile of phage therapy, as it replicates exclusively within bacteria [43], research using mouse models has demonstrated that *Pseudomonas* phages can directly interact with human white blood cells, producing phage RNA and stimulating interferon production. This phenomenon suggests that filamentous phages may interact with the human immune system, potentially exerting direct effects on health [212]. Several preclinical studies have also evaluated phage-induced immune responses. Some of these investigations found that oral phage administration may stimulate inflammatory cytokine production and trigger inflammatory reactions, primarily based on mouse models of intestinal inflammation and dysbiosis [213].

Through research on bacteriophages, scientists have gained deeper insights. Prophages carried by probiotics and their bacterial metabolites may also exert certain negative effects on the outcomes they produce. *Lactobacilli* are indigenous microorganisms in the human gut and are widely used in the food processing industry due to their probiotic properties. Reports indicate that numerous *Lactobacilli* genomes harbor latent phages, which, once released, could contaminate entire batches of fermented products or modulate the gut microbiome. Therefore, comprehensive screening for phages within *Lactobacilli* is crucial for safety assessment and application development of probiotic candidate strains [214]. Furthermore, compared to healthy controls, patients with IBD typically exhibit reduced levels of *Faecalibacterium prausnitzii* in their gut microbiota, while certain phages targeting this bacterium show higher prevalence or abundance in patients, suggesting potential activation of these phages during disease progression. This finding further suggests that phages may trigger or exacerbate *F. prausnitzii* depletion in affected individuals [215].

Bacteriophages demonstrate potential applications in treating infections caused by drug-resistant bacteria, with their specificity and lytic capabilities offering possibilities for targeted antimicrobial therapy. Concurrently, their role in regulating the composition and function of the gut microbiota has drawn attention, potentially providing adjunctive therapeutic effects in scenarios such as *C. difficile* infections. However, potential interactions between phages and the host immune system, long-term effects on microbial balance, and safety concerns regarding their application require more careful evaluation in future research.

### Live dietary microorganisms

It has long been confirmed that there are significant differences between the microbial communities of industrialized and traditional populations, and industrialized societies have a higher prevalence of metabolic, inflammatory, and cardiovascular diseases. Factors such as widespread antibiotic use, diets deficient in complex carbohydrates and live microorganisms, and residence in artificial built environments have contributed to the development of this distinct, non-traditional microbiota in modern industrialized populations [216]. Although direct evidence linking this altered microbial profile to harm in industrialized hosts remains limited, the hypothesis that a deficiency of live microbes in contemporary diets may adversely affect health has spurred interest in the potential benefits of diets rich in living microorganisms [217].

Growing attention is being directed toward dietary microbes for their ability to modulate immune function, cardiovascular health, and neuropsychological conditions such as anxiety, depression, and autism [218]. Central to these effects is the gut-brain axis, through which intestinal microbiota influence neurological processes. Dietary intake of beneficial live microbes may help sustain a functional microbial community, thereby supporting overall health [219].

To quantify microbial intake, researchers have categorized foods into three tiers based on microbial load: fewer than 10^4^ CFU/g (e.g., pasteurized products), 10^4^–10^7^ CFU/g (e.g., unpeeled fruits and vegetables), and more than 10^4^ CFU/g (e.g., unpasteurized fermented foods and probiotic supplements). While this classification is useful for individual foods, it offers limited insight into total daily microbial intake. Applying the validated Sanders protocol [220], participants can be reclassified into low-, moderate-, and high-intake groups according to their total consumption of live microorganisms from all dietary sources [221].

While the probiotic concept focuses on specific strains administered at specified doses, the live dietary microorganism hypothesis suggests that health benefits may also stem from a broader increase in the intake of diverse, safe live microbes. Current research has not yet clarified the extent to which the taxonomic composition or diversity of these dietary microbes affects health outcomes [222]. For instance, the microbial communities in yogurt differ substantially from those on fresh lettuce. Considerable research remains necessary to clarify these relationships. Should future studies confirm the health benefits associated with diets high in live microbes, dietary guidelines such as the U.S. Food Pyramid may be updated to include recommendations for undercooked fermented foods, fruits, and vegetables. Although the current classification of “Live Dietary Microorganisms” lacks a detailed description of microbial types and abundances—deviating from strict biological categorization—documented health benefits could justify the inclusion of microbially rich foods within future biotherapeutic frameworks [223].

## Microbiome-based therapeutics: from the laboratory to the clinic

Microbiome-directed therapies have rapidly advanced and diversified in clinical application. Many microbiome-related products—including everyday foods containing prebiotics, probiotics, or postbiotics, as well as repurposed drugs—are already approved by the FDA and often exempt from additional preclinical safety assessments. Nonetheless, demonstrating efficacy for each targeted condition requires rigorous validation. While safety data for FMT are well established, its regulatory status varies widely: it is classified as a strictly controlled biological product in countries such as the United States, Canada, and Australia; inconsistently regulated as a therapy in the United Kingdom, France, Germany, and Switzerland; and remains unregulated in Austria, Denmark, Sweden, and Finland [224].

Advancing microbiome therapy pipelines requires consensus on candidate prioritization and standardized preclinical safety and efficacy evaluations. Rodent models continue to serve as primary experimental systems, despite their limited capacity to fully recapitulate human microbial interactions [225]. To improve translational relevance, germ-free (gnotobiotic) mice are often colonized with human microbiota; this approach recently enabled the identification of a T-cell signature associated with successful FMT outcomes and increased bacterial loads [226]. Although immortalized human cell lines are commonly used for initial screening, they fail to emulate the physiological complexity of host–microbe crosstalk. Consequently, organoids and organ-on-a-chip systems are increasingly adopted as more sophisticated and human-relevant validation platforms [227].

**Fig. 8:**
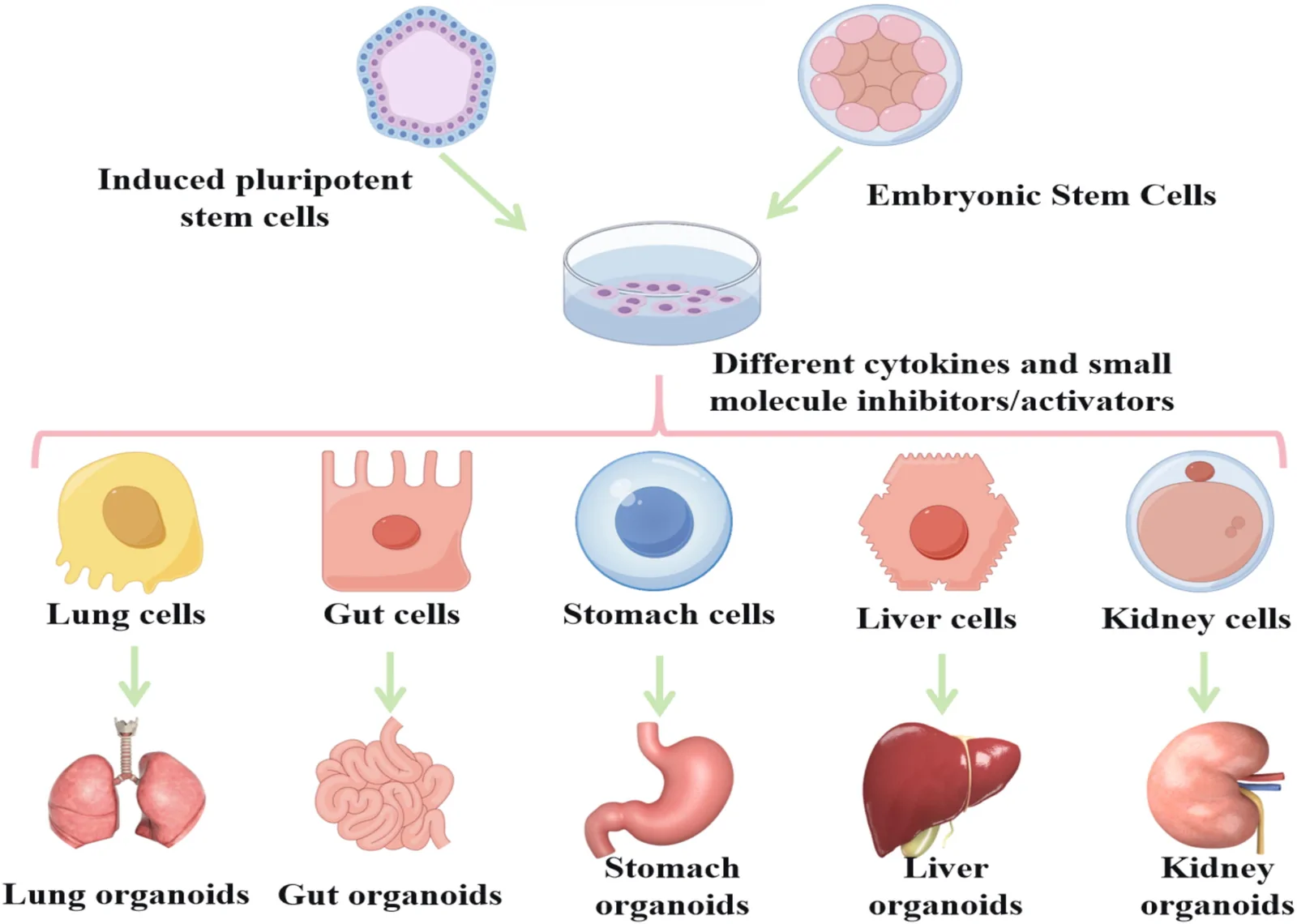
Synthesis of Organoids from Induced Pluripotent Stem Cells.

Organoids are self-organizing three-dimensional (3D) cultures that mimic the multicellular architecture of native tissues [228]. Protocols have been established to generate intestinal organoids from patient-derived cells, partially replicating the *in vivo* microbial niche and enabling personalized microbiome research (Fig. 8). Bacteria can be introduced via microinjection into the organoid lumen to study epithelia-microbial interactions. However, the absence of stromal and vascular components limits the investigation of immune responses [229]. Efforts to enhance complexity—through the use of induced pluripotent stem cell (iPSC)-derived organoids co-cultured with mesenchymal or immune cells—till result enclosed systems that accumulate bacteria, toxic metabolites, and cellular debris [230], [231]. This enclosed environment impedes the evaluation of bacterial colonization, transit dynamics, and immune modulation following treatment with live biotherapeutics. To address these limitations, organ-on-a-chip technologies have been developed.

Organ-on-a-chip systems integrate microfluidics and cell culture to create miniature models of human organs. Platforms mimicking the brain, lung, heart, skin, and gut have already been established [232]. Microchannels enable controlled fluid flow and cyclic mechanical strains that simulate peristalsis, while dual-chamber designs facilitate the establishment of oxygen gradients—anaerobic conditions at the epithelial interface and aerobic conditions in the endothelial/immune cell layer. These systems spontaneously develop villus-like structures and can be populated with either immortalized cell lines or primary tissues, offering flexibility similar to organoids. Researchers have utilized these platforms to study interactions between the host epithelium and immune cells and a variety of microbial agents, including pathogens (e.g., *Shigella, E. coli*), probiotics (e.g., *Lactobacillus*), defined microbial consortia, and even complete personalized microbiomes. However, current chip models do not fully replicate systemic or long-term adaptive immune responses [233]. Further refinement is needed before these platforms can comprehensively assess host-microbiome interactions or reliably evaluate novel therapeutics. Thus, the preclinical assessment of microbiome-based interventions continues to rely on a hybrid approach—combining animal models with advanced *in vitro* systems—prior to clinical trials for efficacy and safety in humans.

Over the past decade, microbiome therapeutics have evolved from simple probiotics and prebiotics to include engineered live biotherapeutics and microbiome-inspired formulations. Progress in culturing gut bacteria and metagenomic sequencing has overcome earlier technical limitations; the current challenge lies in identifying which clinical conditions are most amenable to microbiome modulation and in developing optimal strategies for candidate selection, optimization, and validation. Continued refinement of microbial identification pipelines, preclinical models, and personalized delivery approaches will be essential to advance the field [234].

## Conclusion and future perspective

Research on the human microbiome has successfully evolved from gut-centric exploration into a comprehensive science recognizing that microbial communities are distributed throughout the body and collectively form a dynamic, interconnected functional network. Currently, this field has accumulated sufficient evidence to establish the foundation for clinical translation of a series of microbiome-targeted interventions. Specific probiotic strains, well-validated prebiotics (such as fructooligosaccharides and inulin), carefully designed synbiotic combinations, and postbiotic formulations with defined components have demonstrated clear, reproducible health benefits in managing GI disorders, aiding glycemic and lipid control in T2D patients, and improving specific allergic symptoms through immune modulation. Technologically, biomimetic models such as organoids and organ-on-a-chip can partially replicate complex human microenvironments, providing robust preclinical platforms for screening probiotics, validating postbiotic functions, and investigating host-microbiome interactions in human-relevant systems. These mature tools and interventions collectively provide clinicians with actionable, scientifically grounded strategies for developing personalized nutrition and adjunctive therapies, while equipping the industry with a robust toolkit for developing next-generation functional foods and dietary supplements.

Despite promising prospects, this field still faces a series of profound technical bottlenecks and resource challenges in advancing toward large-scale, precision-targeted clinical and industrial applications. These issues obscure the future development path. First, establishing causality remains a core obstacle; most research remains confined to the level of correlation. Our understanding of how specific bacteria or their metabolites influence host physiology through precise molecular pathways remains fragmented. Second, operational pathways for key technical concepts remain elusive: For instance, how to precisely edit metabolic pathways of specific strains within a community using CRISPR technology, or how to construct mathematical models capable of predicting and mitigating community instability caused by strain competition—these concrete technical roadmaps remain unclear. Similarly, coupled with ingestible microdevices and AI-driven real-time analytics, we are approaching the vision of a “real-time *in vivo* microbiome atlas.” But real-time microbiome mapping remains a conceptual shell, lacking clear definitions of core data dimensions and feasible implementation methods. More tangibly, the large-scale longitudinal cohort studies essential for advancing the field face severe resource constraints. The substantial costs of multi-omics testing, the labor expenses associated with standardized sample collection, and the ethical and legal barriers to cross-border data sharing—these practical challenges are often underestimated behind grand scientific visions, yet they tangibly hinder research depth and breadth. Moreover, the critical issue of dose standardization—unavoidable for any intervention to reach clinical application—remains unsystematically addressed. Significant discrepancies in dose reporting across existing studies make comparative analysis and translational efforts exceedingly difficult.

Looking ahead, to translate the immense potential of microbiome science into universal clinical and real-world benefits, we must undertake a series of focused and pragmatic priority actions. The foremost task is to bridge gaps in technical pathways and definitions: through interdisciplinary collaboration, establish technical standards for “real-time microbiome mapping,” and vigorously develop portable, low-cost sequencing and sensing technologies for low-biomass samples to enable dynamic, affordable monitoring. In mechanism exploration, we should deeply integrate synthetic biology with computational ecology. This involves not only leveraging CRISPR technology for strain-level metabolic engineering but also developing computational models capable of simulating and predicting microbial community dynamics and stability. This will lay the foundation for designing stable, efficient therapeutic microbiomes. Second, resource constraints must be directly addressed to advance solutions: establish international research alliances and data-sharing agreements to unify ethical approval frameworks and reduce compliance costs for cross-border studies. Simultaneously, actively develop and deploy simplified, low-cost targeted detection protocols to replace expensive whole-omics analyses, enabling large-scale, high-frequency monitoring in resource-limited settings. Finally, core bottlenecks in clinical translation must be overcome: Regulatory bodies, academic leaders, and industry experts should jointly establish standardized dosing frameworks and reporting guidelines for microbiome therapies tailored to specific diseases, populations, and strains. Advanced *in vitro* models and miniaturized *in vivo* devices (e.g., ingestible capsules) should be leveraged to establish reliable dose–response relationships. Only through this strategy—combining ambitious scientific visions with pragmatic technical solutions—can microbiome research bridge the gap from laboratory to clinical application, truly becoming an indispensable pillar of precision medicine and preventive healthcare.

**Compliance with ethics requirements**

This article is a comprehensive review of existing scientific literature and does not report any original studies with human participants or animals performed by the authors. Therefore, ethical approval was not required for this work.

**Data availability**

No primary research results have been included and no new data were generated as part of this review.

## CRediT authorship contribution statement

**Chunying Sun:** Conceptualization, Data curation, Methodology, Formal analysis, Visualization, Writing – original draft. **Jingwen Zhu:** Methodology, Writing – review & editing. **Xueyuan Sun:** Methodology, Writing – review & editing. **Zhidong Zhang:** Methodology, Writing – review & editing. **Yantong Sun:** Methodology, Writing – review & editing. **Yan Jin:** Conceptualization, Methodology, Supervision, Writing – review & editing. **Tao Wu:** Conceptualization, Methodology, Supervision, Writing – review & editing.

## Declaration of competing interest

The authors declare that they have no known competing financial interests or personal relationships that could have appeared to influence the work reported in this paper.
